# Evaluation of Systemic Treatments of Small Intestinal Adenocarcinomas: A Systematic Review and Meta-analysis

**DOI:** 10.1001/jamanetworkopen.2023.0631

**Published:** 2023-02-24

**Authors:** Tim de Back, Isabelle Nijskens, Pascale Schafrat, Myriam Chalabi, Geert Kazemier, Louis Vermeulen, Dirkje Sommeijer

**Affiliations:** 1 Center for Experimental and Molecular Medicine, Amsterdam UMC, location University of Amsterdam, Amsterdam, the Netherlands; 2 Oncode Institute, Amsterdam, the Netherlands; 3 Cancer Biology and Immunology, Cancer Center Amsterdam, Amsterdam, the Netherlands; 4 Department of Internal Medicine, Flevohospital, Almere, the Netherlands; 5 Department of Gastrointestinal Oncology, Netherlands Cancer Institute, Amsterdam, the Netherlands; 6 Department of Pathology, Amsterdam UMC, location Vrije Universiteit Amsterdam, Amsterdam, the Netherlands; 7 Department of Surgery, Amsterdam UMC, location Vrije Universiteit Amsterdam, Amsterdam, the Netherlands; 8 Imaging and Biomarkers, Cancer Center Amsterdam, Amsterdam, the Netherlands; 9 Department of Medical Oncology, Amsterdam UMC, location University of Amsterdam, Amsterdam, the Netherlands

## Abstract

**Importance:**

Although small intestinal adenocarcinomas (SIAs) are rare, they have a poor prognosis, and the optimal treatment strategies are largely unknown. Because of the lack of high-quality evidence, guidelines for colorectal cancer are often followed in the treatment of SIAs.

**Objective:**

To review the current evidence regarding survival benefit of systemic therapies, including chemotherapy, targeted agents, and immunotherapy, for patients with SIAs.

**Data Sources:**

Following the Preferred Reporting Items for Systematic Reviews and Meta-analyses, MEDLINE and Embase were searched for articles published from January 1, 2005, until June 1, 2022.

**Study Selection:**

Retrospective cohort studies and prospective phase 2 or 3 trials describing survival after systemic therapies for patients with SIAs were eligible for inclusion. Assessment of study eligibility was blinded and performed by 3 reviewers.

**Data Extraction and Synthesis:**

The reviewers independently extracted data. Random effects, inverse variance, pairwise meta-analyses were performed.

**Main Outcomes and Measures:**

Primary outcomes were overall survival (OS) and progression-free survival (PFS) of patients with SIAs after systemic therapies. Measures of interest included hazard ratios for survival and median survival times.

**Results:**

Overall, 57 retrospective cohort and phase 2 studies of 35 176 patients were included. Adjuvant chemotherapy, generally fluoropyrimidine-based, was associated with increased OS in stage I to III SIAs (hazard ratio [HR], 0.60; 95% CI, 0.53-0.68), especially in stage III tumors (HR, 0.55; 95% CI, 0.48-0.64), irrespective of tumor localization. Palliative chemotherapy was also associated with an OS benefit (HR, 0.48; 95% CI, 0.40-0.58). Fluoropyrimidine-oxaliplatin combinations were superior to other regimens (OS: HR, 0.54; 95% CI, 0.30-0.99; PFS: HR, 0.46; 95% CI, 0.30-0.71). Furthermore, bevacizumab added to chemotherapy compared with chemotherapy alone was associated with significantly prolonged PFS (HR, 0.62; 95% CI, 0.43-0.89). Immunotherapy showed a 50% overall response rate in previously treated defective mismatch repair tumors.

**Conclusions and Relevance:**

In this systematic review and meta-analysis, adjuvant and palliative chemotherapy were both associated with improved survival of patients with SIAs, especially fluoropyrimidine-based regimens and fluoropyrimidine-oxaliplatin combinations. Adding bevacizumab to chemotherapy appears to prolong PFS and deserves further investigation. Immunotherapy seems beneficial and should be considered for patients with defective mismatch repair tumors. International collaborations should be undertaken to confirm and improve efficacy of systemic therapies for patients with SIAs.

## Key Points

**Question:**

Which treatment strategies for small intestinal adenocarcinomas (SIAs) are most beneficial for patient survival?

**Findings:**

In this systematic review and meta-analysis of 57 studies, including 35 176 patients, adjuvant and palliative chemotherapy were associated with improved overall survival irrespective of tumor localization. Palliative fluoropyrimidine-oxaliplatin combinations were superior to other regimens for overall survival and progression-free survival, addition of bevacizumab to chemotherapy was associated with prolonged progression-free survival, and immunotherapy was associated with increased survival for patients with defective mismatch repair tumors.

**Meaning:**

Current evidence supports the clinical use of fluoropyrimidine-oxaliplatin combinations as first-choice chemotherapy for SIAs, addition of bevacizumab to chemotherapy, and immunotherapy for patients with defective mismatch repair SIAs.

## Teaser

This systematic review and meta-analysis explores the survival benefit associated with systemic treatment in patients with small intestinal adenocarcinomas.

## Introduction

Small intestinal adenocarcinomas (SIAs) are rare and poorly characterized. Their incidence varies from 1 to 3.5 per 100 000 inhabitants, despite the considerable size and high epithelial turnover rate of the small intestine.^1,2^ Small intestinal adenocarcinomas have a poor prognosis, with 5-year overall survival (OS) rates of 30.0% to 79.0% for localized disease and 3.0% to 19.0% for metastatic disease.^1,3,4,5,6,7^ This poor prognosis is partly due to diagnostic delay related to vague symptoms. Consequently, one-third of patients with SIAs have metastases at diagnosis.^8,9,10^ In addition, clinical management is extrapolated from colorectal cancer (CRC) studies because of the lack of SIA-specific trials. However, molecular features of SIA differ significantly from CRC, are location specific, and vary according to predisposing disease, which might impact response to therapies and prognosis.^11,12,13,14,15,16,17^

A first international guideline recommends adjuvant capecitabine combined with oxaliplatin (CAPOX); fluorouracil, leucovorin, and oxaliplatin (FOLFOX); or fluoropyrimidine monotherapy.^18^ In the first-line setting, similar agents or triple therapy with fluorouracil, leucovorin, oxaliplatin, and irinotecan (FOLFOXIRI) are suggested. Bevacizumab is not standard in the treatment of SIAs. Immunotherapy is also not standard, despite high efficacy in other defective mismatch repair (dMMR) tumors.^18,19,20,21^ So far, these regimens have not been evaluated in dedicated randomized clinical trials of patients with SIAs.

Overall, the outcome of patients with SIAs is poor, molecular characteristics are unique, and the most effective systemic therapeutic regimens are largely unknown. We aimed to comprehensively summarize the available literature on survival benefit of systemic treatment in patients with SIAs. This effort indicates most beneficial treatment options and could inform future clinical trials to improve outcomes for patients with SIAs.

## Methods

### Study Design

This study was reported according to the Preferred Reporting Items for Systematic Reviews and Meta-analyses (PRISMA) reporting guidelines.^22^ Detailed descriptions of the methods are available in the eMethods in Supplement 1.

The primary objective was to assess the survival benefit of systemic therapies for locoregional and metastatic SIAs,^23^ measured by OS. Secondary outcomes entailed progression-free survival (PFS), relapse-free survival (RFS), and cancer-specific survival (CSS) after systemic therapies for SIA, reported as hazard ratios (HRs) or median survival times in months (median OS [mOS] and median PFS [mPFS]).

### Search Strategy and Study Selection

MEDLINE and Embase were searched for studies published between January 1, 2005, and June 1, 2022, on the survival benefit of systemic therapies for patients with SIAs. Eligible studies contained HRs, survival times, or response rates after systemic therapies. Studies on ampullary adenocarcinomas (AACs), surgery, or radiotherapy were excluded. Study selection was performed by 3 reviewers independently (T.d.B., I.N., and P.S.).

### Quality Assessment and Data Extraction

Consensus-based assessment of risk of bias, using the Cochrane Risk of Bias in Non-randomized Studies of Interventions (ROBINS-I) tool,^24^ and data extraction were performed by 3 independent reviewers (T.d.B, I.N., and P.S.). Studies with high risk of bias were not excluded. Missing data were requested from the authors of the original studies.

### Statistical Analysis

Meta-analyses were performed separately for the adjuvant and palliative setting with HRs for OS, RFS, PFS, and CSS and with mOS and mPFS data. For each meta-analysis, subsets of the included studies were selected depending on reported data and line of therapy. We performed random-effects, inverse variance, pairwise meta-analyses. To compare 4 first-line regimens, network meta-analyses (NMAs) were performed using the GeMTC platform.^25^

Heterogeneity was assessed with the Higgins *I*^2^ index and the Cochran *Q* test; heterogeneity was considered present if an *I*^2^ was 50.0% or greater or a Cochrane *Q* test *P* ≤ .05. Studies that contributed to heterogeneity in a meta-analysis were excluded in sensitivity analyses to assess their effect on the estimated outcome. In addition, subgroup analyses and metaregression for geography, risk of bias, publication period, adjustment of HRs, and stage and line of therapy were run to identify potential causes for heterogeneity. Nonheterogeneous effect estimates were reported. Publication bias was assessed with funnel plots and the Egger test.^26^

All analyses were performed with R software, version 4.0.5 (R Foundation for Statistical Computing) and the GeMTC platform, version 1.0-1.^25^ Hypothesis tests were 2-sided, with a significance level of *P* < .05.

## Results

### Selection of Eligible Studies

The search yielded 4353 studies, of which 116 were eligible for further review (eFigure 1 in Supplement 1). After full-text revision, 57 studies were included, with a total of 35 176 patients. Among the 57 studies, 6 studies^27,28,29,30,31,32^ were prospective cohort studies or phase 2 trials, and 51 studies^1,3,4,17,19,33,34,35,36,37,38,39,40,41,42,43,44,45,46,47,48,49,50,51,52,53,54,55,56,57,58,59,60,61,62,63,64,65,66,67,68,69,70,71,72,73,74,75,76,77,78^ were retrospective in nature (Table; eTables 1 and 2 in Supplement 1). Thirty studies^1,3,4,17,27,33,34,35,36,37,38,39,40,41,42,43,44,45,46,47,48,49,50,51,52,53,54,55,56,57^ assessed survival after adjuvant chemotherapy, and 40 studies^1,3,4,17,19,27,28,29,30,32,33,35,37,39,44,46,48,57,58,59,60,61,62,63,64,65,66,67,68,69,70,71,72,73,74,75,76,77,78,79^ described benefit of palliative chemotherapeutics. Thirteen studies^1,3,4,17,27,33,35,37,39,44,46,48,57^ were included in both groups. Depending on data availability, subsets of the total number of 57 studies were selected for each meta-analysis (eFigures 2-12 in Supplement 1). Missing data were requested for 28 articles. Three studies^37,67,68^ were excluded from subanalyses because missing data could not be obtained.

**Table.** Overview of Included Studies

Characteristic	No. (%) of studies	
	Adjuvant therapy (n = 30)	Palliative therapy (n = 40)
Study design		
Retrospective	29 (96.7)	34 (85.0)
Prospective	1 (3.3)	6 (15.0)
Study origin		
North America	13 (43.3)	15 (37.5)
Asia	9 (30)	12 (30.0)
Europe	8 (26.7)	13 (32.5)
Disease stages		
Stage I	3 (10.0)	NA
Stage II	4 (13.3)	NA
Stage III	7 (23.3)	NA
Stage II-III	2 (6.7)	NA
Stage 0-III/I-III	23 (76.7)	NA
Locally advanced	NA	1 (2.5)
Stage IV	NA	39 (97.5)
Unknown	1 (3.3)	0
Location		
All	18 (60.0)	37 (92.5)
Duodenum	11 (36.7)	12 (30.0)
Jejunum-ileum	3 (10.0)	3 (7.5)
Survival outcomes		
Overall	25 (83.3)	39 (97.5)
Progression free	3 (10.0)	23 (57.5)
Relapse free	12 (40.0)	0
Cancer specific	1 (3.3)	3 (7.5)
Systemic therapy		
Chemotherapy	16 (53.3)	26 (65.0)
Fluoropyrimidine-based	14 (46.7)	14 (35.0)
Combined with irinotecan	0	8 (20.0)
Fluoropyrimidine monotherapy	2 (6.7)	3 (7.5)
S1-based^a^	2 (6.7)	0
Targeted therapy	NA	7 (17.5)
Bevacizumab	NA	5 (12.5)
Anti-EGFR	NA	3 (7.5)
Pembrolizumab	NA	1 (2.5)
Not specified	14 (46.7)	14 (35.0)
Adjuvant	30 (100)	NA
First line	NA	12 (30.0)
Second line	NA	6 (15.0)
Third line	NA	2 (5.0)
Second and third lines	NA	1 (2.5)

Abbreviations: EGFR, epidermal growth factor receptor; NA, not applicable.

^a^ Tegafur, gimeracil, and oteracil.

### Overview of Study Characteristics and Risk of Bias

Most studies describing adjuvant chemotherapy focused on all stages (I-III) (n = 26) and all localizations (n = 18) (Table). Sixteen studies^17,27,36,37,38,40,42,44,45,46,48,49,53,55,56,57^ specified the type of adjuvant chemotherapy. Most adjuvant chemotherapy was fluoropyrimidine-based. Studies in the palliative setting commonly studied all SIA localizations. Twenty-six studies^3,17,27,28,29,30,32,33,35,44,46,48,58,59,60,61,62,63,64,68,70,72,74,75,76,77^ (65.0%) specified the chemotherapy regimens. Targeted agents were investigated in 7 studies,^31,60,61,63,64,71,76^ of which most assessed bevacizumab.

Of 57 studies, 23 studies^3,17,27,29,30,31,32,33,36,38,44,49,50,51,58,60,62,63,70,71,73,74,77^ (40.4%) were subject to serious risk of bias due to the lack of multivariable analyses and selection bias (eTable 3 in Supplement 1). Thirty-three studies^1,4,19,28,34,35,37,39,40,41,42,43,45,46,47,48,52,53,54,55,56,57,59,61,64,65,66,67,69,72,75,76,78^ (57.9%) had medium or low risk of bias. For 1 study,^68^ risk of bias was not assessable because information was lacking. In general, selection bias is a concern in our analyses.

### Adjuvant Chemotherapy

**Figure 1.**
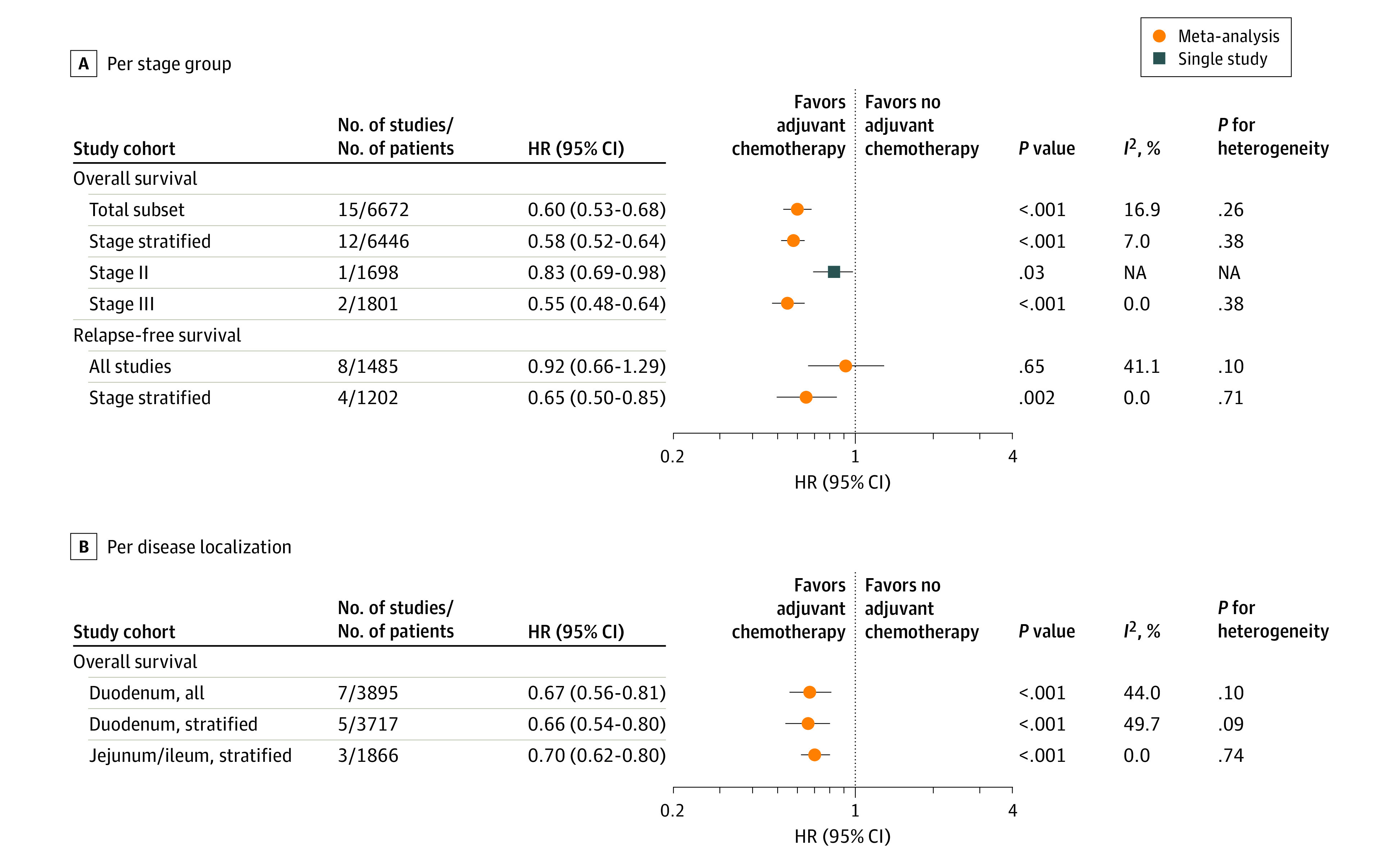
Overall Survival and Relapse-Free Survival Benefit of Adjuvant Chemotherapy per Stage Group and per Localization HR indicates hazard ratio; NA, not applicable.

We assessed the benefit of adjuvant chemotherapy vs no systemic treatment in stage I to III SIAs across all localizations (Figure 1A). Covering 15 retrospective cohorts with a total of 6672 patients,^1,4,33,34,38,39,40,43,45,47,49,51,52,53,56^ we found that adjuvant chemotherapy was associated with OS benefits (HR, 0.60; 95% CI, 0.53-0.68). When we selected stage-stratified studies (n = 12 studies; 6446 patients), OS benefit was similar (HR, 0.58; 95% CI, 0.52-0.64). Adjuvant chemotherapy was associated with prolonged OS mainly in stage III tumors (HR, 0.55; 95% CI, 0.48-0.64), although patients with stage II disease also benefitted (HR, 0.83; 95% CI, 0.69-0.98).

Subgroup analyses and metaregression were performed in the total subset of 15 studies^1,4,33,34,38,39,40,43,45,47,49,51,52,53,56^ based on geography, publication date, risk of bias, adjustment of HRs, and stage, to assess their association with the OS benefit of adjuvant chemotherapy (eTables 4 and 5 in Supplement 1). None of these parameters were significantly associated with OS after therapy.

We also assessed RFS after adjuvant chemotherapy (Figure 1A). When the original studies stratified for stage, we observed a significantly longer RFS after adjuvant chemotherapy (HR, 0.65; 95% CI, 0.50-0.85). However, this was not significant without stratification for stage (HR, 0.92; 95% CI, 0.66-1.29), suggesting that RFS benefit after adjuvant treatment is stage dependent. Regarding localization, adjuvant chemotherapy was equally associated with OS benefits of duodenal (HR, 0.67; 95% CI, 0.56-0.81) and jejunal or ileal (HR, 0.70; 95% CI, 0.62-0.80) adenocarcinomas (Figure 1B). Four retrospective studies^35,36,38,54^ reported mOS after adjuvant chemotherapy, which was pooled 51.9 (95% CI, 45.7-58.1) months vs 28.0 (95% CI, 23.0-32.9) months when untreated compared with 21.8 to 50.6 months vs 8.6 to 28.2 months in studies^42,43,44^ that could not be pooled.

### Palliative Chemotherapy

**Figure 2.**
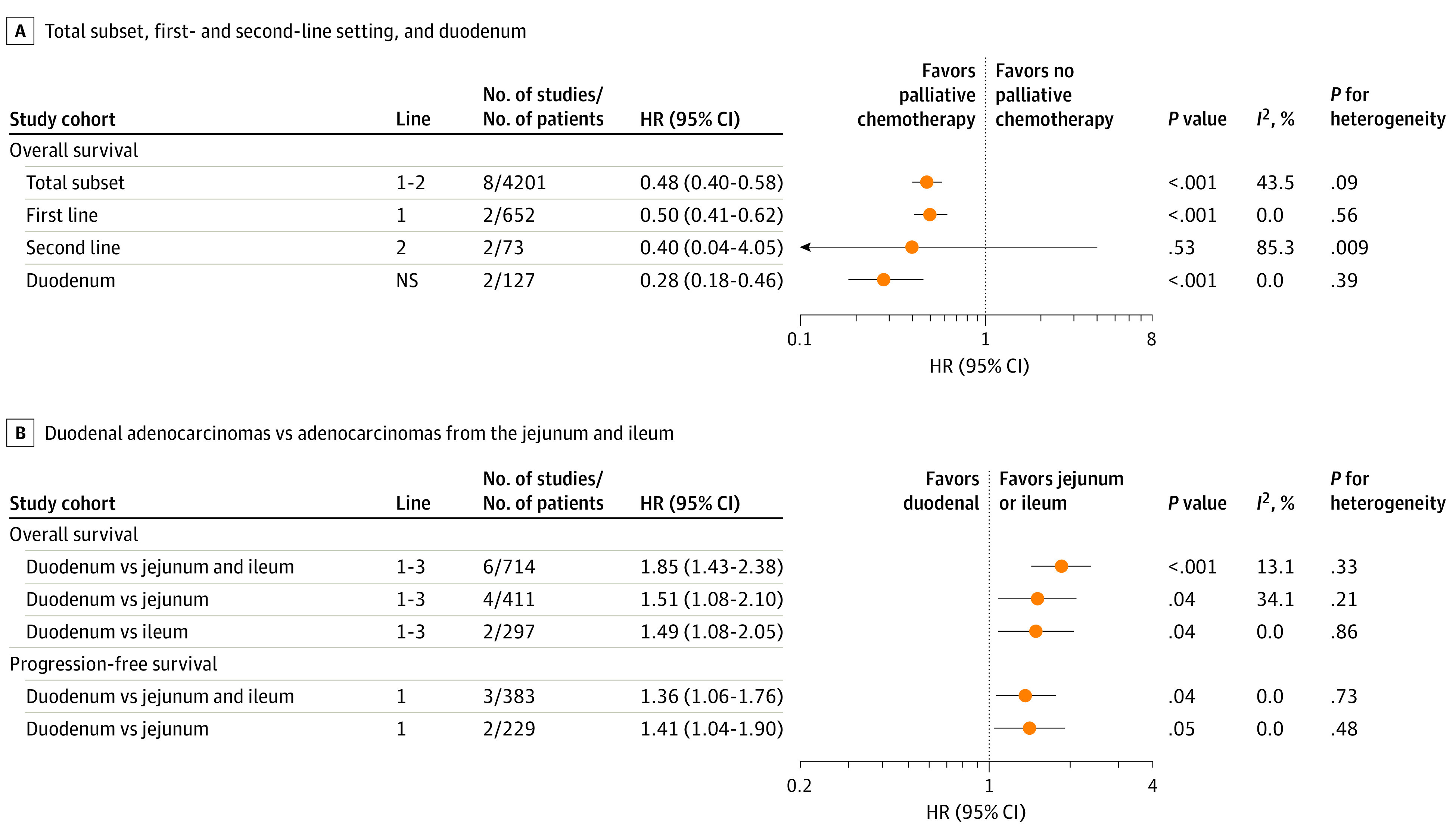
Association of Palliative Chemotherapy With Overall Survival Benefit for the Total Subset, in the First- and Second-line Settings, and for Duodenal Adenocarcinomas Alone and With Overall Survival and Progression-Free Survival After Palliative Chemotherapy for Duodenal Adenocarcinomas Compared With Adenocarcinomas From Jejunum and Ileum HR indicates hazard ratio; NS, not specified.

Multiple-line palliative chemotherapy was associated with improved OS over no chemotherapy in a subset of 1 prospective study^27^ and 7 retrospective studies^1,4,35,59,65,69,78^ (4201 patients with metastatic SIA; HR, 0.48; 95% CI, 0.40-0.58) (Figure 2A). The same was found for CSS in 2 studies^69,78^ with 448 patients (HR, 0.62; 95% CI, 0.49-0.80). In the first line, chemotherapy was associated with increased OS (HR, 0.50; 95% CI, 0.41-0.62), whereas this outcome was unclear for the second line (HR, 0.40; 95% CI, 0.04-4.05). For duodenal adenocarcinomas separately, palliative chemotherapy also improved OS (HR, 0.28; 95% CI, 0.18-0.46). When comparing palliatively treated duodenal vs distal adenocarcinomas, the latter consistently had better OS and PFS (Figure 2B).

Subgroup analyses and metaregression in the subset of 8 studies^1,4,27,35,59,65,69,78^ were performed to evaluate the association of geography, publication date, risk of bias, adjustment of HRs, and line of therapy with OS benefit after palliative chemotherapy (eTables 6 and 7 in Supplement 1). None of these variables were significantly associated with the estimated benefit from palliative chemotherapy.

**Figure 3.**
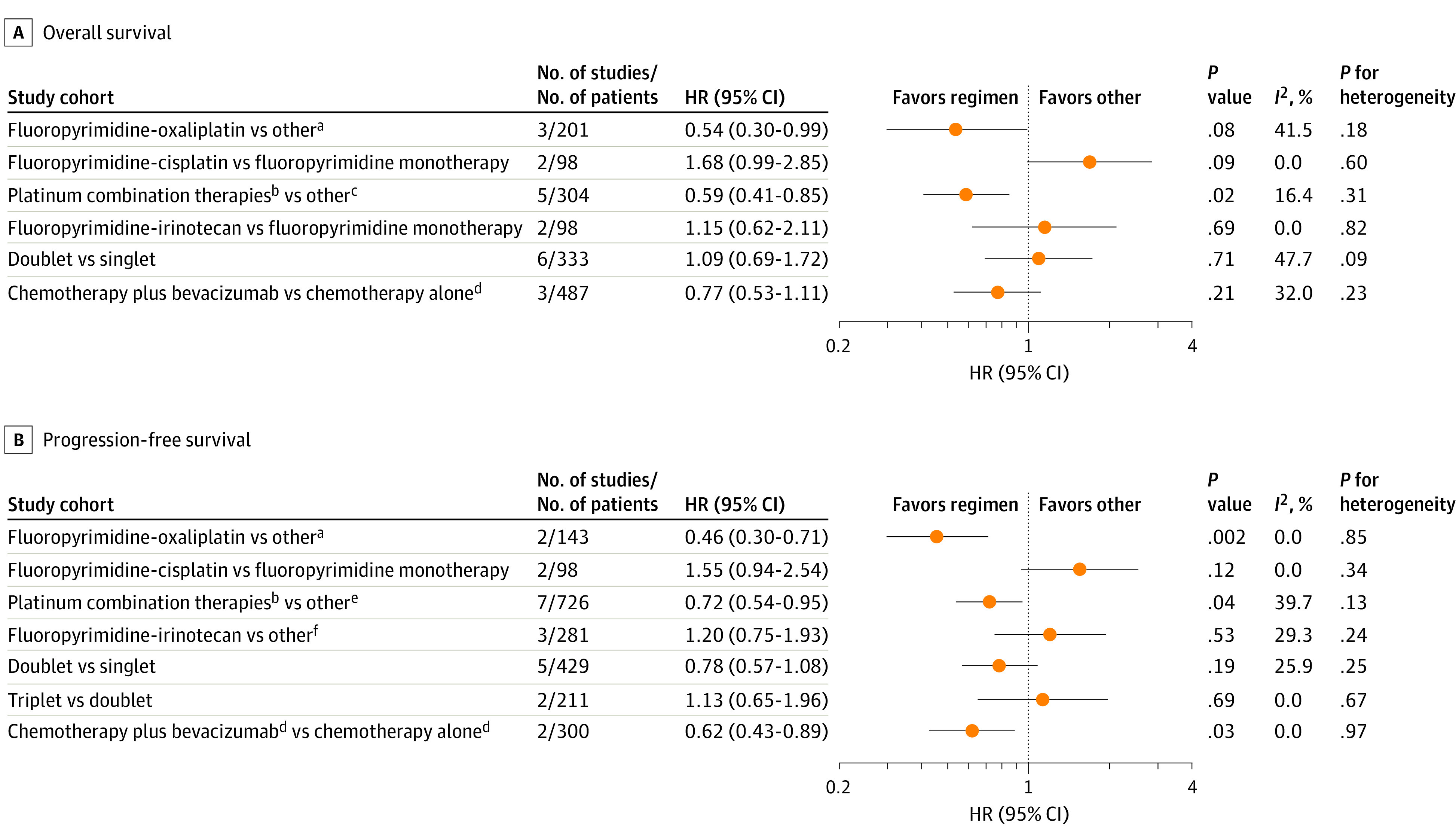
Comparison of Associations of First-line Palliative Chemotherapy Regimens With Overall Survival and Progression-Free Survival HR indicates hazard ratio. ^a^Fluoropyrimidine monotherapy and fluoropyrimidine cisplatin. ^b^Combinations with oxaliplatin, carboplatin, and cisplatin. ^c^Fluoropyrimidine monotherapy, platinum monotherapy, nonplatinum combination, and nonplatinum or nonfluoropyrimidine therapies. ^d^Predominantly platinum combination and fluoropyrimidine monotherapy. ^e^Fluoropyrimidine monotherapy, platinum monotherapy, nonplatinum combination, nonplatinum or nonfluoropyrimidine therapies, and folinic acid, fluorouracil, and irinotecan. ^f^Fluoropyrimidine monotherapy and platinum combination.

Next, we compared different first-line regimens on OS and PFS (Figure 3). Platinum combinations (oxaliplatin, carboplatin, and cisplatin) were highly beneficial. Specifically, the fluoropyrimidine-oxaliplatin combination was associated with prolonged survival (OS: HR, 0.54; 95% CI, 0.30-0.99; PFS: HR, 0.46; 95% CI, 0.30-0.71), whereas fluoropyrimidine-cisplatin appeared unfavorable (OS: HR, 1.68; 95% CI, 0.99-2.85); PFS: HR, 1.55; 95% CI, 0.94-2.54). Fluoropyrimidine-irinotecan was not superior to other regimens (OS: HR, 1.15; 95% CI, 0.62-2.11; PFS HR, 1.20; 95% CI, 0.75-1.93). Doublets seemed to be associated with improved PFS compared with singlets but not with prolonged OS. Triplets were not associated with improved PFS compared with doublets.

Regarding bevacizumab, the addition of bevacizumab to chemotherapy was associated with significantly improved PFS compared with chemotherapy alone (HR, 0.62; 95% CI, 0.43-0.89) (Figure 3B). Overall survival seemed to improve as well, although nonsignificantly (HR, 0.77; 95% CI, 0.53-1.11) (Figure 3A). Chemotherapy backbones were fluoropyrimidine-platinum, fluoropyrimidine monotherapy, and fluoropyrimidine-irinotecan in both arms.

**Figure 4.**
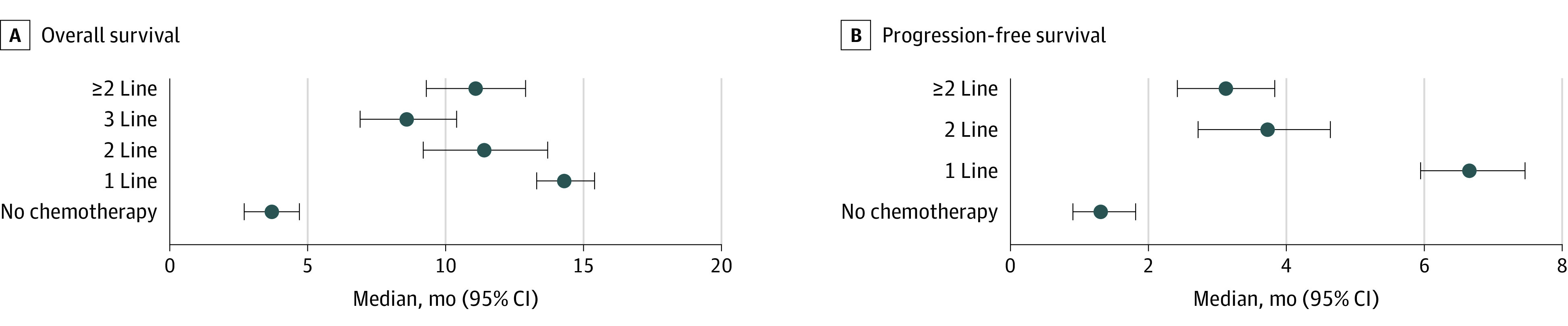
Median Survival in Patients With Metastatic Small Intestinal Adenocarcinomas Whiskers indicate 95% CIs.

After first-line chemotherapy, the mOS was 14.5 (95% CI, 13.5-15.5) months and the mPFS was 6.7 (95% CI, 6.0-7.4) months, decreasing to an mOS of 8.6 (95% CI, 6.9-10.4) months in the third line (Figure 4). In nonpooled studies,^1,3,19,59,62,66,71,72^ the mOS after first-line chemotherapy ranged from 6.8 to 22.2 months vs 2.0 to 12.0 months for untreated patients. In line with our previous meta-analyses, the mPFS was best after FOLFOX therapy (8.2 months; 95% CI, 7.0-9.3 months) and fluoropyrimidine-oxaliplatin therapy (including CAPOX) (7.5 months; 95% CI, 6.0-9.0 months). Worse mPFS was observed after fluoropyrimidine-irinotecan (6.1 months; 95% CI, 4.7-7.5 months) or fluoropyrimidine-cisplatin (6.6 months; 95% CI, 5.4-7.8 months).

To indirectly compare 4 first-line regimens, we performed NMAs (eFigure 13 in Supplement 1). Similar to our pairwise meta-analyses, fluoropyrimidine-oxaliplatin was most associated with OS benefit compared with fluoropyrimidine monotherapy (HR, 0.64; 95% CI, 0.30-1.44), fluoropyrimidine-irinotecan (HR, 0.56; 95% CI, 0.19-1.75), and fluoropyrimidine-cisplatin (HR, 0.38; 95% CI, 0.13-1.14). Fluoropyrimidine-cisplatin was consistently associated with worse OS. The same was found for PFS. Fluoropyrimidine-irinotecan seemed more favorable than fluoropyrimidine-cisplatin but worse compared with fluoropyrimidine-oxaliplatin. As shown in eFigure 14 in Supplement 1, the pairwise meta-analyses and NMAs gave identical results, indicating robustness of the NMAs.

Response rates to palliative chemotherapy were consistent with our previous analyses (eTable 2 in Supplement 1). Overall response rates for fluoropyrimidine-oxaliplatin varied from 34.0% to 56.0%.^28,32,59,72,75^ For fluoropyrimidine-irinotecan, the overall response rates ranged from 9.0% to 55.0%,^48,59,61,72,75^ and for fluoropyrimidine-cisplatin, the overall response rates ranged from 31.0% to 38.0%.^59,72,75^ For triplet regimens, overall response rates of 83.0% (folinic acid, fluorouracil, and irinotecan-oxaliplatin) and 38.0% (capecitabine, irinotecan, and oxaliplatin) were reported.^29,61^

### Heterogeneity, Publication Bias, and Certainty of Evidence

Eleven of our 31 pairwise meta-analyses pooling HRs and 7 of our 15 meta-analyses with medians were heterogeneous. eTables 8 and 9 in Supplement 1 demonstrate correction of heterogeneity in sensitivity analyses. Heterogeneity was partially explained by publication date and adjustment of HRs in the palliative setting (eTable 7 in Supplement 1), differences in cohort sizes, and treatment regimens. Specifically, inclusion of cisplatin doublets caused heterogeneity in the meta-analyses of platinum combinations vs other regimens. After exclusion of outlier studies, heterogeneity was resolved.

In our NMAs, heterogeneity was limited, as observed with the random-effects SDs (OS: SD, 0.30; range, 0.02-0.76; PFS: SD, 0.36; range, 0.02-0.76), which were both smaller than the effect sizes in the models. No outliers or influential studies were detected when assessing residual deviance and leverage statistics.

Of our 46 meta-analyses, 2 were found to have publication bias (eFigures 15-20 in Supplement 1). These were the meta-analyses on the OS benefit of second-line chemotherapy compared with no treatment and on mPFS after second- and third-line palliative chemotherapy. The results of these analyses should be carefully interpreted.

Certainty of evidence of all meta-analyses was assessed using the Grading of Recommendations, Assessment, Development, and Evaluations (GRADE) tool (eTables 10 and 11 in Supplement 1).^80^ Eleven of our meta-analyses scored very low, 12 scored low, 21 scored moderate, and 2 scored high.

### Prospective Evaluation of Targeted Agents and Immunotherapy

One study^20^ prospectively assessed CAPOX with bevacizumab in 23 patients with SIA and 7 patients with AAC and found an mOS of 12.9 months, an mPFS of 8.7 months, and an overall response rate of 48.3%, suggesting that CAPOX with bevacizumab is an active regimen for advanced SIA. On the contrary, panitumumab did not improve survival in 9 patients with *RAS* (OMIM 606614) wild-type tumors (including 1 patient with AAC), with an mOS of 5.7 months and an mPFS of 2.4 months.^81^

Regarding immunotherapy, the anti–programmed cell death ligand 1 antibody avelumab was tested in 8 patients (including 3 patients with AAC), without selection for dMMR status, and revealed an mPFS of 8.0 months.^82^ Pembrolizumab, targeting programmed cell death 1, was administered to 40 patients with SIAs and found an overall response rate of 50% in the subgroup of 4 patients with dMMR tumors.^79^

## Discussion

This meta-analysis aimed to summarize prospective and retrospective data on the survival benefit of systemic therapies for patients with SIAs. Our meta-analyses show benefit from adjuvant chemotherapy regardless of localization and increasing by incremental disease stage. Furthermore, chemotherapy extends OS and PFS in the palliative setting. Consistently, first-line fluoropyrimidine-oxaliplatin was most favorable for survival in both pairwise meta-analyses and NMAs. Addition of bevacizumab to first-line chemotherapy significantly improved PFS compared with chemotherapy alone. Finally, immunotherapy seemed to prolong survival for patients with dMMR tumors.

Previous studies^1,4,9,27,33,39,40,43,45,47,50,51,54,83^ have been inconclusive with regard to benefit from adjuvant chemotherapy. Our meta-analyses are in line with large retrospective cohort studies^1,4,47,54^ that all observed survival benefit of adjuvant chemotherapy compared with surgery alone, with HRs ranging from 0.55 to 0.83. However, some earlier studies^9,27,33,39,40,43,45,50,51,83^ could not prove benefit of adjuvant chemotherapy, perhaps because of small cohort sizes, the lack of correction for prognostic variables, or the inclusion of chemoradiation.

Studies to refine clinical applicability of adjuvant chemotherapy are needed for several reasons. First, selection of patients with SIAs for adjuvant treatment remains unclear. For CRC, high-risk patients with stage II or stage III disease are eligible, but it is unknown whether these selection criteria also apply to patients with SIAs. In addition, patients with duodenal adenocarcinomas are at higher risk of being unfit for adjuvant chemotherapy after Whipple surgery compared with patients with distal adenocarcinomas. Second, specific regimens need to be compared in randomized clinical trials to determine most effective therapies. For this purpose, a first international phase 3 trial was set up (Global BALLAD [A Trial to Evaluate the Potential Benefit of Adjuvant Chemotherapy for Small Bowel Adenocarcinoma]) to compare observation alone, monotherapy with fluoropyrimidines, and FOLFOX for patients with stage I to III SIAs.^84^ Simultaneously, a phase 3 trial has been initiated in Japan (J-BALLAD) in which observation alone will be compared with CAPOX.^85^ Third, tailoring therapy to molecular tumor traits requires further attention, for example, (neo-)adjuvant immunotherapy for patients with localized dMMR SIAs.

Only a few studies in patients with metastatic SIAs assessed survival benefit of palliative chemotherapy. In most studies,^7,28,32,59,72,75,86^ fluoropyrimidine-oxaliplatin was superior to other regimens in terms of survival and response rates. This finding is perhaps not unexpected because this regimen is also highly effective in other gastrointestinal cancers.^87,88^ For cisplatin combinations, our study suggested a smaller survival benefit compared with other regimens. The 2 studies^59,74^ that demonstrated a higher mOS and mPFS for cisplatin compared with oxaliplatin combinations included mainly duodenal adenocarcinomas (80%), which might be sensitive to cisplatin-based treatment due to their anatomical proximity to the pancreas and stomach.^89,90^

Targeted agents are rarely used in patients with SIAs because of unclear efficacy. Our results do not allow for any definitive conclusions on this matter. However, addition of bevacizumab to chemotherapy appeared to be more beneficial for survival than chemotherapy alone. This finding is in line with trials performed in metastatic CRC.^91^ For anti–epidermal growth factor receptor therapy, hardly any data are available. The available data do not support clinical activity, even in patients with *RAS* wild-type tumors.^63,81^ This finding might be related to the reduced activity seen in the right side of the colon, which develops from the same embryonic structure as the small intestine (midgut).

Regarding immunotherapy, evidence shows its activity in varying solid dMMR cancers, with response rates ranging from 38.0% to 50.0% and even long-term survival.^21,79,92,93^ These data indicate that immune checkpoint inhibition should be the preferred treatment choice in dMMR metastatic SIAs over bevacizumab.

### Limitations

Our meta-analysis is affected by several limitations. Unfortunately, most studies^1,3,4,17,19,33,34,35,36,37,38,39,40,41,42,43,44,45,46,47,48,49,50,51,52,53,54,55,56,57,58,59,60,61,62,63,64,65,66,67,68,69,70,71,72,73,74,75,76,77,78^ were nonrandomized cohort studies. Hence, the outcomes of some of our meta-analyses are subject to selection bias and confounding by prognostic factors. The calculated survival benefit of chemotherapy administration, compared with no chemotherapy, is likely overestimated because of routine selection of fitter patients for systemic therapy. In our view, this limitation plays a lesser role in the meta-analyses that compare different treatment regimens because selection bias stemming from patient performance or disease burden is likely more limited in this case. Furthermore, with subgroup and metaregression analyses, we confirmed that risk of bias did not significantly impact the results of our meta-analyses. In addition, the use of both adjusted and unadjusted HRs did not significantly impact outcomes, as shown in our metaregression analyses. However, differences in adjustment variables could introduce bias, although the variables adjusted for were relatively consistent across studies. Furthermore, the scarcity of data precluded most pairwise regimen comparisons. Similarly, the numbers of eligible studies for the NMAs were limited. Therefore, our NMAs solely serve as an enforcement of the findings in this meta-analysis. Additionally, data were missing for some studies, which led to exclusion of studies in the meta-analyses pooling medians. Publication bias did not affect most of our meta-analyses.

## Conclusions

In this systematic review and meta-analysis, adjuvant fluoropyrimidine-based chemotherapy was associated with a survival benefit for patients with SIAs, irrespective of disease localization. Benefit increased by incremental stage, providing a 17.0% vs 45.0% reduction of mortality hazard for stage II and III disease, respectively. For metastatic SIA, first-line fluoropyrimidine-oxaliplatin combinations were associated with the largest survival benefit compared with other regimens. Addition of bevacizumab to chemotherapy and immunotherapy for the treatment of dMMR tumors seemed beneficial but requires further investigation. Our results could be used to guide future clinical trials into the most effective treatment regimens, patient selection for adjuvant chemotherapy, and efficacy of targeted agents and immunotherapy for patients with SIAs.

## Notes

Supplement 1.
eMethods.
**eTable 1.** Overview of studies describing adjuvant chemotherapy **eTable 2.** Overview of studies describing palliative chemotherapy **eTable 3.** Risk of bias assessment using the Cochrane ROBINS-I tool **eTable 4.** Subgroup analyses for adjuvant chemotherapy versus no chemotherapy **eTable 5.** Metaregression for adjuvant chemotherapy versus no chemotherapy **eTable 6.** Subgroup analyses for palliative chemotherapy versus no chemotherapy **eTable 7.** Metaregression for palliative chemotherapy versus no chemotherapy **eTable 8.** Sensitivity analyses for meta-analyses with hazard ratios **eTable 9.** Sensitivity analyses for meta-analyses with median survival data **eTable 10.** Certainty of evidence score for meta-analyses with hazard ratios **eTable 11.** Certainty of evidence score for meta-analyses with median survival data **eFigure 1.** PRISMA flowchart of study selection **eFigure 2.** Forest plots **eFigure 3.** Forest plots **eFigure 4.** Forest plots **eFigure 5.** Forest plots **eFigure 6.** Forest plots **eFigure 7.** Forest plots **eFigure 8.** Forest plots **eFigure 9.** Forest plots **eFigure 10.** Forest plots **eFigure 11.** Forest plots **eFigure 12.** Forest plots **eFigure 13.** Network meta-analyses for first-line regimens **eFigure 14.** Comparison of pairwise and network meta-analyses **eFigure 15.** Evaluation of publication bias **eFigure 16.** Evaluation of publication bias **eFigure 17.** Evaluation of publication bias **eFigure 18.** Evaluation of publication bias **eFigure 19.** Evaluation of publication bias **eFigure 20.** Evaluation of publication bias
eReferences

Supplement 2.
Data Sharing Statement
