# Supplementary material for: Evaluation of Systemic Treatments of Small Intestinal Adenocarcinomas: A Systematic Review and Meta-analysis
Source: JAMA Netw Open. 2023 Feb 24;6(2):e230631. doi: 10.1001/jamanetworkopen.2023.0631 (PMC9958532; doi:10.1001/jamanetworkopen.2023.0631)
Supplement: Supplement 1. — eMethods. eTable 1. Overview of studies describing adjuvant chemotherapy eTable 2. Overview of studies describing palliative chemotherapy eTable 3. Risk of bias assessment using the Cochrane ROBINS-I tool eTable 4. Subgroup analyses for adjuvant chemotherapy versus no chemotherapy eTable 5. Metaregression for adjuvant chemotherapy versus no chemotherapy eTable 6. Subgroup analyses for palliative chemotherapy versus no chemotherapy eTable 7. Metaregression for palliative chemotherapy versus no chemotherapy eTable 8. Sensitivity analyses for meta-analyses with hazard ratios eTable 9. Sensitivity analyses for meta-analyses with median survival data eTable 10. Certainty of evidence score for meta-analyses with hazard ratios eTable 11. Certainty of evidence score for meta-analyses with median survival data eFigure 1. PRISMA flowchart of study selection eFigure 2. Forest plots eFigure 3. Forest plots eFigure 4. Forest plots eFigure 5. Forest plots eFigure 6. Forest plots eFigure 7. Forest plots eFigure 8. Forest plots eFigure 9. Forest plots eFigure 10. Forest plots eFigure 11. Forest plots eFigure 12. Forest plots eFigure 13. Network meta-analyses for first-line regimens eFigure 14. Comparison of pairwise and network meta-analyses eFigure 15. Evaluation of publication bias eFigure 16. Evaluation of publication bias eFigure 17. Evaluation of publication bias eFigure 18. Evaluation of publication bias eFigure 19. Evaluation of publication bias eFigure 20. Evaluation of publication bias eReferences [file jamanetwopen-e230631-s001.pdf]

Supplemental Online Content

de Back T, Nijskens I, Schafrat P, et al. Evaluation of systemic treatments of small intestinal adenocarcinomas: a systematic review and meta-analysis. *JAMA Netw Open*. 2023;6(2):e230631. doi:10.1001/jamanetworkopen.2023.0631

eMethods.

eTable 1. Overview of studies describing adjuvant chemotherapy

eTable 2. Overview of studies describing palliative chemotherapy

eTable 3. Risk of bias assessment using the Cochrane ROBINS-I tool

eTable 4. Subgroup analyses for adjuvant chemotherapy versus no chemotherapy

eTable 5. Metaregression for adjuvant chemotherapy versus no chemotherapy

eTable 6. Subgroup analyses for palliative chemotherapy versus no chemotherapy

eTable 7. Metaregression for palliative chemotherapy versus no chemotherapy

eTable 8. Sensitivity analyses for meta-analyses with hazard ratios

eTable 9. Sensitivity analyses for meta-analyses with median survival data

eTable 10. Certainty of evidence score for meta-analyses with hazard ratios

eTable 11. Certainty of evidence score for meta-analyses with median survival data

eFigure 1. PRISMA flowchart of study selection

eFigure 2. Forest plots

eFigure 3. Forest plots

eFigure 4. Forest plots

eFigure 5. Forest plots

eFigure 6. Forest plots

eFigure 7. Forest plots

eFigure 8. Forest plots

eFigure 9. Forest plots

eFigure 10. Forest plots

eFigure 11. Forest plots

eFigure 12. Forest plots

eFigure 13. Network meta-analyses for first-line regimens

eFigure 14. Comparison of pairwise and network meta-analyses

eFigure 15. Evaluation of publication bias

eFigure 16. Evaluation of publication bias

eFigure 17. Evaluation of publication bias

eFigure 18. Evaluation of publication bias

eFigure 19. Evaluation of publication bias

eFigure 20. Evaluation of publication bias

eReferences

This supplemental material has been provided by the authors to give readers additional information about their work.

© 2023 de Back T et al. *JAMA Network Open.*

eMethods

Study protocol

The PROSPERO international prospective register of systematic reviews was consulted before the start of the study and no ongoing reviews with a similar scope were identified. Our study protocol was submitted to PROSPERO to undergo review before the start of the literature search and is accessible via the PROSPERO database (ID: CRD42020202395).[1]

Search strategy and study selection

We conducted a literature search in Medline (via PubMed) and Embase to retrieve original studies and conference abstracts on the efficacy of systemic therapies in SIA from 2005 until June 2022. Case reports were excluded from the search. Key words were systemic therapies, chemotherapy, targeted therapies, immunotherapy and small intestinal adenocarcinoma. Eligible studies contained survival data, either absolute survival times in months or hazard ratios (HRs), or response rates for systemic therapies in SIA patients. Studies including ampullary adenocarcinomas (AACs) were excluded. All variants of systemic therapy were deemed eligible. Studies focusing on surgery and radiotherapy were excluded. Overlap in cohorts between studies was accounted for by excluding the smaller studies. Both study selection based on title and abstract and full text screening were performed by three reviewers independently (TB, IN, PS) using Rayyan software, a web and mobile application for systematic reviews.[2] After removal of blinding, the selected studies were discussed and inclusion of the studies was consensus-based. No disagreements occurred.

Quality assessment and data extraction

Risk of bias was assessed for all included original reports by three reviewers independently (TB, IN, PS) using Cochrane's ROBINS-I tool.[3] Studies were classified into low, median, serious or critical risk of bias. Assessment was consensus-based and no disagreements occurred. Studies were not excluded based on high risk of bias.
Conference abstracts and full-text manuscripts, including supplementary materials, were screened for data of interest. Data extraction was performed by three reviewers independently (TB, IN, PS) and any inconsistencies were discussed and resolved by consensus. We collected data on general study characteristics, such as study design, number of included patients, study country, relevant exclusion criteria, SIA localization, stage and median follow-up. Data of interest consisted of treatment setting (adjuvant or palliative), therapy regimen, line of therapy, number of patients in treated and control groups, survival endpoint, median absolute overall survival (mOS) and progression-free survival (mPFS) with 95% confidence interval (95% CI), HRs for overall survival (OS), progression-free survival (PFS), relapse-free survival (RFS) and cancer-specific survival (CSS) with 95% CI and *P* value, and response rates. HRs preferably were adjusted, though unadjusted HRs were extracted when multivariable Cox regression analyses were not available. Independent variables used in multivariable analyses were documented.

Missing data

Relevant missing data were requested from the authors of the original studies.
For two studies, the 5-year OS probability, the log-rank *P* value and the number of events were used to approximate the HR for OS. To this end, we used the notion that the log-rank statistic follows an approximately standard normal distribution with mean ln(HR) sqrt(n*d/4), where ln(HR) denotes the natural logarithm of the HR and the product n*d is the expected number of events in the analysis. It follows that ln(HR) can be approximated by ζ_α sqrt(4/D), where D is the observed number of events and ζ_α is the upper α quantile of the standard normal distribution that corresponds to the log-rank *P* value. The standard error (SE) of the ln(HR) was estimated by exploiting the notion that the log-rank statistic is equivalent to the score test for the Cox proportional hazards model. Hence, the log-rank *P* value is asymptotically similar to the *P* value for the ln(HR) from that model. The 95% CI of the HR could then be calculated with the corresponding SE.
Missing *P* values and 95% CIs of hazard ratios were calculated using previously published methods.[4,5]

© 2023 de Back T et al. *JAMA Network Open.*

Statistical analysis
Due to the range of survival outcomes and comparisons described in the original articles, the numbers of studies per meta-analysis are fractions of the total number of studies included in this report. Per meta-analysis, eligible studies were selected upfront, depending on the available data. Meta-analyses were performed with both hazard ratios and median survival times. In the adjuvant setting, we compared (m)OS and RFS of patients treated with adjuvant chemotherapy versus no chemotherapy, also per stage and per disease localization. In the palliative setting, (m)OS, (m)PFS and CSS of patients treated with palliative chemotherapy were compared to untreated patients, and also per line of therapy and per disease localization. Furthermore, pairwise comparisons of palliative regimens in the first line were performed for (m)OS and (m)PFS. Lastly, network meta-analyses (NMAs) were performed to indirectly compare OS and PFS of four first-line regimens.
Exclusion of studies per meta-analysis was predominantly based on heterogeneity (See "Evaluation and handling of heterogeneity in meta-analyses" and eTables 8 and 9 in the Supplement). A small fraction of studies was excluded from the sub-analyses with median survival times due to missing data.
We performed random-effect, inverse variance pairwise meta-analyses, using the R packages "meta" and "metafor".[6] The DerSimonian-Laird τ^2-estimator was used to pool HRs, whereas the Restricted Maximum-Likelihood τ^2-estimator was used to pool median survival times.
Random-effect NMAs were conducted using the GeMTC platform.[7] Robustness of the NMA was controlled with the run length and convergence, assessed by the Potential Scale Reduction Factor.
All analyses were performed with R software version 4.0.5 and the GeMTC platform, version 1.0-1.[7] Median absolute survival data were described as medians with 95% CI and response data as percentages of the total. Survival comparisons were described with HRs, 95% CI and *P* value and plotted as forest plots. *P* values were two-sided, with a significance level of $P < .05$. Multiple testing correction was performed by applying the classical Benjamini-Hochberg procedure per individual hypothesis (i.e., independently for the analyses into adjuvant chemotherapy and palliative chemotherapy), using the R package "multtest".[8]

Evaluation and handling of heterogeneity in meta-analyses
Between-study heterogeneity in pairwise meta-analyses was assessed using the Higgins I^2 index and the Cochrane's Q-test; heterogeneity was considered present if an I^2 ≥50.0% or a Cochrane's Q-test *P* value ≤.05. Heterogeneity was explored by identifying outliers and influential studies, followed by sensitivity analyses. Studies that were found to be outliers or influential, were excluded from the meta-analyses in sensitivity analyses in order to correct for heterogeneity. Additionally, subgroup analyses and meta-regression were carried out to identify drivers of heterogeneity, using the R packages "dmetar" and "meta".[6] Subgroup analyses and meta-regression were performed in the largest meta-analyses of the adjuvant and palliative setting to explore the effect of geography, study period, bias level, adjustment of HRs, stage and line of therapy on the heterogeneity level and estimated outcomes. We reported the non-heterogeneous pooled estimates after heterogeneity exploration. Heterogeneous pooled estimates were shown in the Supplement.
Heterogeneity in the NMAs was explored by calculating the random-effect standard deviation (SD) of the model and compared to the estimated effect sizes per treatment regimen. Additionally, effect sizes from the NMAs were compared to the effect sizes of pairwise meta-analyses, including the same studies. The NMAs were not suitable for node-splitting due to the limited studies included.

Publication bias
Publication bias was explored with funnel plots and quantified with the Egger's test.[9]
Publication bias was considered present when the Egger's test was significant or evident asymmetry occurred in the funnel plots.

Certainty of evidence assessment of meta-analyses
The Grading of Recommendations, Assessment, Development and Evaluations (GRADE) tool was used to assess certainty of evidence of each meta-analysis.[10] Certainty of evidence was scored very low, low, moderate or high, depending on the risk of bias, imprecision of the effect size, inconsistency of studies, indirectness of evidence and publication bias. If more than 50% of studies in a meta-analysis had low or serious risk of bias, GRADE risk of bias was scored accordingly. Otherwise, meta-analyses were scored as being at medium risk of bias. Possible publication bias was scored if meta-analyses showed asymmetry of the funnel plot in the absence of an Egger's test. Furthermore, if a meta-analysis that pooled less than four studies scored 'high' on GRADE assessment, the score was downgraded with one level to 'moderate' due to the small cohort size.

© 2023 de Back T et al. *JAMA Network Open.*

eTable 1. Overview of studies included in the adjuvant setting

										Localization based on total N in study					
Study	Country	Design	N[a]	N[b]	Stages	Stage I N (%)	Stage II N (%)	Stage III N (%)	Stage NOS N (%)	Duodenum N (%)	Jejunum N (%)	Ileum N (%)	NOS N (%)	Regimens*	Outcome
Akce et al,[11] 2019	USA	R	7954	1419	II-III	-	506 (35.7)	913 (64.3)	-	4607 (57.9)	1241 (15.6)	857 (10.8)	1249 (15.7)	NS	OS
Aparicio et al,[12] 2020	France	P	347	110	0-III	NS	NS	NS	NS	208 (60.6)	71 (20.7)	64 (18.7)	4 (1.2)	F-based	OS
Aparicio et al,[13] 2013	France	R	63	34	I-III	NS	NS	NS	NS	32 (50.8)	18 (28.6)	13 (20.6)	-	5-FU based	RFS
De Jong et al,[14] 2022	Netherlands	R	585	94	I-III	NS	NS	NS	NS	585 (100)	-	-	-	NS	OS
Duerr et al,[15] 2016	Canada	R	150	29	I-III	NS	NS	NS	NS	72 (48.0)	46 (30.7)	32 (21.3)	-	F-based	OS, RFS
Ecker et al,[16] 2016	USA	R	2297	1142	I-III	35 (3.0)	485 (42.5)	622 (54.5)	-	827 (36.0)	988 (43.0)		482 (21.0)	NS	OS
Fishman et al,[17] 2006	Canada	R	113	15	III	-	-	15 (100)	-	80 (70.8)	19 (16.8)	9 (8.0)	5 (4.4)	NS	OS, TTP
Guo et al,[18] 2014	China	R	119	36	I-III	7 (19.4)	17 (47.2)	10 (27.8)	2 (5.6)	111 (93.3)	6 (5.0)	2 (1.7)	-	mFOLFOX6; CAPOX	OS
Hong et al,[19] 2009	South-Korea	R	53	13	I-III	NS	NS	NS	NS	39 (73.6)	7 (13.2)	7 (13.2)	-	5-FU based	OS
Huffman et al,[20] 2019	USA	R	241	85	I-III	4 (4.7)	25 (29.4)	56 (65.9)	-	156 (64.7)	56 (23.2)	22 (9.1)	7 (2.9)	FOLFOX; Various	OS
Jensen et al,[21] 2022	Denmark	R	96	45	I-III	NS	NS	NS	NS	96 (100)	-	-	-	F-based	OS
Kaslow et al,[22] 2022	USA	R	2956	1158	I-III	83 (7.2)	199 (17.2)	241 (20.8)	635 (54.8)	2956 (100)	-	-	-	NS	OS
Khan et al,[23] 2015	UK	R	84	48	I-III	NS	NS	NS	NS	70 (65.4)	21 (19.6)	15 (14.0)	1 (2.1)	NS	OS, RFS
Kim et al,[24] 2014	South-Korea	R	50	21	I-III	NS	NS	NS	NS	50 (100)	-	-	-	5-FU based	OS
Koo et al,[25] 2011	South-Korea	R	52	23	I-III	-	10 (43.5)	13 (56.5)	-	34 (65.4)	18 (34.6)		-	F-based	OS, RFS
Lee et al,[26] 2020	USA	R	7019	1069	I-III	155 (14.5)	432 (40.4)	482 (45.1)	-	3767 (53.7)	3252 (46.3)		-	NS	OS
Legué et al,[27] 2016	Netherlands	R	1775	392	I-III	-	91 (23.2)	248 (63.3)	53 (13.5)	1026 (57.8)	336 (18.9)	257 (14.5)	156 (8.8)	NS	OS
Li et al,[28] 2020	China	R	148	55	I-III	NS	NS	NS	NS	112 (75.7)	6 (4.1)	22 (14.9)	8 (5.4)	F-based	OS, RFS
Liang et al,[29] 2012	Taiwan	R	36	8	I-III	NS	NS	NS	NS	36 (100)	-	-	-	NS	OS, RFS
Mohammed et al,[30] 2015	USA	R	269	68	I-III	NS	NS	38 (55.9)	NS	120 (44.6)	-	-	149 (55.4)	NS	OS
Moon et al,[31] 2010	South-Korea	R	100	16	I-III	NS	NS	9 (56.3)	NS	82 (82.0)	11 (11.0)	7 (7.0)	-	F-based	OS, RFS
Nakagawa et al,[32] 2022	Japan	R	1083	278	I-III	NS	NS	NS	NS	1083 (100)	-	-	-	S-1-based	RFS
Overman et al,[33] 2010	USA	R	54	30	II-III	-	11 (37.0)	19 (63.0)	-	36 (66.7)	11 (20.4)	7 (13.0)	-	F-based	OS, RFS
Overman et al,[34] 2008	USA	R	80	8	I-III	NS	NS	NS	NS	30 (37.5)	35 (43.8)	6 (7.5)	9 (11.3)	5-FU based	OS, PFS
Platoff et al,[35] 2020	USA	R	2487	1067	0-III	NS	NS	NS	NS	2487 (100)	-	-	-	NS	OS
Sakaguchi et al,[36] 2022	Japan	R	47	20	II-III	-	NS	NS	-	47 (100)	-	-	-	S-1-based	OS, RFS
Solaini et al,[37] 2015	UK	R	150	68	I-III	NS	NS	NS	NS	150 (100)	-	-	-	NS	OS, RFS
Yanko et al,[38] 2022	Canada	R	112	18	I-III	NS	NS	NS	NS	53 (47)	17 (15)	26 (23)	16 (14)	F-based	OS, RFS
Young et al,[39] 2016	USA	R	2123	207	I-III	NS	NS	NS	NS	-	632 (29.8)	708 (33.4)	783 (36.9)	Various	OS, CSS
Zaanan et al,[40] 2011	France	R	28	7	NS	NS	NS	NS	NS	12 (42.9)	16 (57.1)		-	F-based; Oxali-based	OS, PFS

Abbreviations: N, number of patients; NOS, not otherwise specified; R, retrospective; NS, not specified; OS, overall survival; P, prospective; F-based, fluoropyrimidine-based; RFS, relapse-free survival; TTP, time to progression; mFOLFOX6, modified fluorouracil, leucovorin and oxaliplatin; CAPOX, capecitabine and oxaliplatin; CSS, cancer-specific survival; Oxali-based, oxaliplatin-based.
[a]Total number of patients per study; [b]Total number of patients treated with adjuvant chemotherapy per study; *All studies included a control group not treated with adjuvant chemotherapy.

© 2023 de Back T et al. *JAMA Network Open.*

eTable 2. Overview of studies included in the palliative setting

Study	Country	Design	N[a]	N[b]	Localization based on total N in study<br>Duodenum N (%)	Jejunum N (%)	Ileum N (%)	NOS N (%)	Line	Treatment regimens	ORR	Outcome
Amano et al,[41] 2021	Japan	R	74	74	38 (51.3)	27 (36.5)	9 (12.2)	-	1	Platinum+bev<br>Platinum-based	-<br>-	OS, PFS
Akce et al,[11] 2019	USA	R	7954	1636	4607 (57.9)	1241 (15.6)	857 (10.8)	1249 (15.7)	NS	NS*	-	OS
Aldrich et al,[42] 2019	USA	R	20	20	10 (50.0)	10 (50.0)		-	NS	Taxane-based	30.0%	OS, TTP
Aparicio et al,[12] 2020	France	P	347	86	208 (60.6)	71 (20.7)	64 (18.7)	4 (1.2)	NS	Various*	-	OS
Aparicio et al,[13] 2013	France	R	63	41	32 (50.8)	18 (28.6)	13 (20.6)	-	NS	F-based*	-	OS
Aydin et al,[43] 2016	Turkey	R	71	56	55 (77.5)	7 (9.9)	9 (12.7)	-	1	mFOLFOX6*;<br>FOLFIRI;<br>Cisplatin-5-FU;<br>Gemcitabine	56.0%<br>55.0%<br>35.0%<br>20.0%	OS, PFS
Aydin et al,[44] 2017	Turkey	R	28	28	16 (57.1)	7 (25.0)	5 (17.9)	-	1	mFOLFOX6/FOLFIRI<br>+ bevacizumab;<br>mFOLFOX6/<br>FOLFIRI	58.0%<br><br>44.0%	OS, PFS
Bhamidipati et al,[45] 2021	USA	R	437	296	215 (49.0)	142 (32.5)	70 (16.0)	10 (2.3)	1, 2 and 3	F-platinum;<br>F-mono;<br>FOLFIRI;<br>FOLFIRINOX;<br>Various	59.0%<br>48.0%<br>46.0%<br>83.0%<br>-	OS, TTP
Czaykowski et al,[46] 2007	Canada	R	47	37	18 (38.3)	15 (31.9)	9 (19.1)	6 (12.8)	NS	F-based*	-	OS
De Jong et al,[47] 2022	Netherlands	R	167	115	167 (100.0)	-	-	-	1	F-based	-	OS
Dell'Aquila et al,[48] 2020	Italy	R	13	13	4 (30.8)	4 (30.8)	5 (38.5)	-	1 and 2	CTx +/- cetuximab	58.0%	OS, PFS
Duerr et al,[15] 2016	Canada	R	150	33	72 (48.0)	46 (30.7)	32 (21.3)	-	NS	Pyrimidine-based*	-	OS
Fishman et al,[17] 2006	Canada	R	113	44	80 (70.8)	19 (16.8)	9 (8.0)	5 (4.4)	1, 2, and 3	Various*	-	OS, TTP
Hirao et al,[49] 2017	Japan	R	27	27	8 (29.6)	11 (40.7)	8 (29.6)	-	1	Various	-	OS, PFS
Hong et al,[19] 2009	South-Korea	R	53	15	39 (73.6)	7 (13.2)	7 (13.2)	-	NS	NS*	-	OS
Horimatsu et al,[50] 2017	Japan	P	24	22	14 (58.3)	10 (17.2)	0 (0.0)	-	1	mFOLFOX6	45.0%	OS, PFS
Khan et al,[23] 2015	UK	R	59	46	40 (67.8)	11 (18.6)	8 (13.6)	-	1 and 2	Various*	-	OS, PFS
Koo et al,[51] 2011	South-Korea	R	91	40	71 (78.0)	20 (22.0)		-	NS	F-based*	-	OS, PFS
Legué et al,[27] 2016	Netherlands	R	1775	581	1026 (57.8)	336 (18.9)	257 (14.5)	156 (8.8)	1	NS*	-	OS
Legué et al,[52] 2019	Netherlands	R	522	199	345 (66.1)	81 (15.5)	56 (10.7)	40 (7.7)	1, 2 and 3	Various	-	OS, TTP
Legué et al,[53] 2017	Netherlands	R	181	57	80 (44.2)	40 (22.1)	41 (22.7)	20 (11.0)	NS	NS*	-	OS
Legué et al,[54] 2019	Netherlands	R	187	187	112 (59.9)	38 (20.3)	23 (12.3)	14 (7.5)	1	CTx +/- bevacizumab	-	OS
Liu et al,[55] 2014	China	R	56	56	56 (100.0)	-	-	-	NS	F-based*	-	OS, PFS
Liu et al,[56] 2020	USA	R	1219	700	788 (64.6)	163 (13.4)	93 (7.6)	175 (14.4)	NS	NS*	-	OS, CSS
McWilliams et al,[57] 2017	USA	P	33	33	19 (57.6)	10 (30.3)	3 (9.1)	1 (3.0)	1	CAPIRINOX	38.0%	OS, PFS
Moon et al,[31] 2010	South-Korea	R	100	34	82 (82.0)	11 (11.0)	7 (7.0)	-	1 and 2	Various*	-	OS, PFS
Nakazawa et al,[58] 2020	Japan	R	27	21	13 (48.1)	14 (51.9)	0 (0.0)	-	2	Irinotecan-based*;<br>Taxane-based	-<br>-	OS, PFS
Overman et al,[59] 2018	USA	P	13	13	4 (30.8)	9 (69.2)			≥2	Nab-paclitaxel	15.0%	OS, PFS
Overman et al,[34] 2008	USA	R	80	80	30 (37.5)	35 (43.8)	6 (7.5)	9 (11.3)	1	F-platinum;<br>Various	46.0%	OS, PFS

© 2023 de Back T et al. *JAMA Network Open.*

Pedersen et al,[60] 2019	USA	P	40	40	24 (60.0)	10 (25.0)	6 (15.0)	-	≥2	Pembrolizumab	8.0%	OS, PFS
Takayoshi et al,[61] 2017	Japan	R	33	33	21 (70.0)	7 (23.3)	1 (3.3)	1 (3.3)	≥2	Bevacizumab +/- CTx	-	OS
Tsushima et al,[62] 2012	Japan	R	132	132	80 (60.6)	52 (39.4)		-	1	F-mono;<br>F-cisplatin;<br>F-oxaliplatin;<br>F-irinotecan	20.0%<br>38.0%<br>42.0%<br>25.0%	OS, PFS
Xiang et al,[63] 2012	China	P	33	33	26 (78.8)	7 (21.2)		-	1	mFOLFOX6	49.0%	OS, PFS
Yanko et al,[38] 2022	Canada	R	112	39	53 (47.0)	17 (15.0)		26 (23.0)	NS	F-based*	-	OS, RFS
Ye et al,[65] 2020	USA	R	506	230	NS	NS	NS	NS	NS	NS*	-	CSS
Yhim et al,[66] 2015	South-Korea	R	58	58	50 (86.2)	8 (13.8)		-	1	F-cisplatin;<br>F-oxaliplatin;<br>F-irinotecan;<br>F-mono	-<br>-<br>-<br>-	OS, PFS
Young et al,[39] 2016	USA	R	2123	517	-	632 (29.8)	708 (33.4)	783 (36.9)	NS	NS*	-	OS, CSS
Zaanan et al,[66] 2010	France	R	93	93	55 (59.1)	38 (40.9)		-	1	F-mono;<br>FOLFOX;<br>FOLFIRI;<br>F-cisplatin	0.0%<br>34.0%<br>9.0%<br>31.0%	OS, PFS
Zaanan et al,[40] 2011	France	R	28	51	12 (42.9)	16 (57.1)		-	2	FOLFIRI;<br>FOLFOX;<br>F-cisplatin	20.0%<br>-<br>-	OS, PFS
Zhu et al,[67] 2022	USA	R	373	263	156 (41.8)	217 (58.2)		-	NS	NS*	-	OS, CSS

Abbreviations: N, number of patients; NOS, not otherwise specified; ORR, overall response rate; R, retrospective; bev, bevacizumab; OS, overall survival; PFS, progression-free survival; NS, not specified; TTP, time to progression; P, prospective; F-, fluoropyrimidine; mFOLFOX6, modified fluorouracil, leucovorin and oxaliplatin; FOLFIRI, fluorouracil, leucovorin, irinotecan; CTx, chemotherapy; CSS, cancer-specific survival; CAPIRINOX, capecitabine, irinotecan, oxaliplatin.
[a]Total number of patients per study; [b]Total number of stage IV patients treated with palliative chemotherapy per study; *Included a control group not treated with palliative chemotherapy.

© 2023 de Back T et al. *JAMA Network Open.*

eTable 3. Risk of bias assessment with Cochrane's ROBINS-I tool

Study	Domains							Overall	Setting	
	Confounding	Selection bias	Classification of interventions	Reporting bias	Deviations from interventions	Missing data	Measuring outcomes	RoB	Adjuvant	Palliative
Akce et al,[11] 2019	L	M	M	M	L	M	L	M	Y	Y
Amano et al,[41] 2022	L	M	M	L	L	L	M	L		Y
Aldrich et al,[42] 2019	S	M	M	L	L	M	M	S		Y
Aparicio et al,[12] 2020	S	L	L	L	L	L	M	S	Y	Y
Aparicio et al,[13] 2013	S	S	M	M	L	M	L	S	Y	Y
Aydin et al,[43] 2016	L	M	L	L	L	L	M	L		Y
Aydin et al,[44] 2017	S	M	L	S	L	L	M	S		Y
Bhamidipati et al,[45] 2021	M	M	L	M	L	M	M	M		Y
Czaykowski et al,[46] 2007	S	M	L	S	L	L	M	S		Y
De Jong et al,[47] 2022	S	S	L	L	L	L	M	S		Y
De Jong et al,[14] 2022	L	M	M	L	L	L	M	L	Y	
Dell'Aquila et al,[48] 2020	S	M	L	L	L	L	M	S		Y
Duerr et al,[15] 2016	S	M	L	L	L	M	M	S	Y	Y
Ecker et al,[16] 2016	L	M	M	L	L	M	M	M	Y	
Fishman et al,[17] 2006	M	M	L	L	L	M	M	M	Y	Y
Guo et al,[18] 2014	S	M	L	L	L	L	M	S	Y	
Hirao et al,[49] 2017	L	M	L	L	L	M	M	L		Y
Hong et al,[19] 2009	S	M	L	L	L	M	M	M	Y	Y
Horimatsu et al,[50] 2017	L	L	L	L	L	L	M	L		Y
Huffman et al,[20] 2019	S	M	L	L	L	M	M	S	Y	
Jensen et al,[21] 2022	L	M	L	L	L	L	M	L	Y	
Kaslow et al,[22] 2022	L	L	M	L	L	M	M	L	Y	
Khan et al,[23] 2015	M	M	M	L	L	M	M	M	Y	Y
Kim et al,[24] 2014	S	M	L	L	L	M	M	S	Y	
Koo et al,[51] 2011	L	M	L	L	L	L	M	L		Y
Koo et al,[25] 2011	L	M	L	L	L	M	M	L	Y	
Lee et al,[26] 2020	L	M	M	L	L	M	M	M	Y	

© 2023 de Back T et al. *JAMA Network Open.*

eTable 3. (continuation)

Study	Domains: Confounding	Domains: Selection bias	Domains: Classification of interventions	Domains: Reporting bias	Domains: Deviations from interventions	Domains: Missing data	Domains: Measuring outcomes	Overall RoB	Setting: Adjuvant	Setting: Palliative
Legué et al,[27] 2016	L	M	M	L	L	M	L	L	Y	Y
Legué et al,[52] 2019	L	M	L	L	L	M	M	L		Y
Legué et al,[53] 2017	L	M	M	L	L	L	M	L		Y
Legué et al,[54] 2019	L	M	L	L	L	L	M	L		Y
Li et al,[28] 2020	L	M	L	L	L	L	M	L	Y	
Liang et al,[29] 2012	S	M	M	L	L	L	M	S	Y	
Liu et al,[55] 2014	NI	NI	M	L	L	NI	M	NI		Y
Liu et al,[56] 2020	L	M	M	M	L	M	M	M		Y
McWilliams et al,[57] 2017	S	L	L	L	L	L	M	S		Y
Mohammed et al,[30] 2015	L	M	M	L	L	M	M	M	Y	
Moon et al,[31] 2010	S	M	L	L	L	M	M	S	Y	Y
Nakagawa et al,[32] 2022	L	M	L	L	L	M	M	L	Y	
Nakazawa et al,[58] 2020	S	M	L	L	L	NI	M	S		Y
Overman et al,[59] 2018	S	M	L	L	L	S	M	S		Y
Overman et al,[33] 2010	L	M	L	L	L	M	M	L	Y	
Overman et al,[34] 2008	L	M	L	M	L	M	M	M	Y	Y
Pedersen et al,[60] 2019	S	M	L	L	L	NI	M	S		Y
Platoff et al,[35] 2020	L	M	M	L	L	M	M	M	Y	
Sakaguchi et al,[36] 2022	L	M	L	L	L	NI	M	L	Y	
Solaini et al,[37] 2015	S	M	M	L	L	S	M	S	Y	
Takayoshi et al,[61] 2017	S	M	L	L	L	NI	M	S		Y
Tsushima et al,[62] 2012	L	M	L	L	L	M	M	L		Y
Xiang et al,[63] 2012	S	L	L	L	L	L	M	S		Y
Yanko et al,[38] 2022	L	M	L	L	L	L	M	L	Y	Y
Ye et al,[64] 2020	L	M	M	L	L	S	M	S		Y
Yhim et al,[65] 2015	S	S	L	L	L	M	M	S		Y
Young et al,[39] 2016	S	M	M	L	L	L	M	S	Y	Y

eTable 3. (continuation)

© 2023 de Back T et al. *JAMA Network Open.*

Study	Domains							Overall	Setting	
	Confounding	Selection bias	Classification of interventions	Reporting bias	Deviations from interventions	Missing data	Measuring outcomes	RoB	Adjuvant	Palliative
Zaanan et al,[66] 2010	M	M	L	L	L	M	M	M		Y
Zaanan et al,[40] 2011	L	M	L	L	L	L	M	L	Y	Y
Zhu et al,[67] 2022	L	M	L	L	L	L	M	L		Y

Abbreviations: RoB, risk of bias; Y, yes.
Risk of bias assessment: L = low risk of bias, M = medium risk of bias, S = serious risk of bias, C = critical risk of bias (not used), NI = no information.

© 2023 de Back T et al. *JAMA Network Open.*

eTable 4. Subgroup analyses for adjuvant chemotherapy versus no treatment

Meta-analyses	Original values<br>HR (95% CI)	Subgroups<br>Group (n)	Values subgroup analyses<br>HR (95% CI)	Difference between subgroups<br>*P* value
Adj CTx vs no OS	0.60 (0.53 to 0.68), *P*<.001	Asia (n=3)	0.50 (0.25 to 0.99), *P*=.05	0.62
Geography		Europe (n=5)	0.68 (0.47 to 1.00), *P*=.05	
		North America (n=7)	0.58 (0.52 to 0.64), *P*<.001	
Adj CTx vs no OS	0.60 (0.53 to 0.68), *P*<.001	<2015 (n=3)	0.57 (0.36 to 0.90), *P*=.16	0.75
Publication date		2015-2020 (n=8)	0.64 (0.53 to 0.77), *P*< .001	
		2020-present (n=4)	0.53 (0.33 to 0.87), *P*=.01	
Adj CTx vs no OS	0.60 (0.53 to 0.68), *P*<.001	Low (n=6)	0.55 (0.39 to 0.79), *P*<.001	0.69
Risk of bias		Medium (n=5)	0.58 (0.53 to 0.64), *P*<.001	
		Serious (n=4)	0.70 (0.45 to 1.10), *P*=.13	

Abbreviations: HR, hazard ratio; 95% CI, 95% confidence interval; n, number of studies; adj CTx, adjuvant chemotherapy; OS, overall survival

© 2023 de Back T et al. *JAMA Network Open.*

eTable 5. Meta-regression analysis for adjuvant chemotherapy versus no chemotherapy overall survival

Variable	Original value<br>HR (95% CI)	Subgroups<br>Group (n)	Regression coefficients<br>Estimates	Predicted HRs<br>HR (95% CI)	Explained heterogeneity<br>I^2 (%)	Test of moderator<br>*P* value
Geography	0.60 (0.53 to 0.68), *P* <.001	Intercept: Asia (n=3)	-0.64	0.53 (0.31 to 0.88)	0.0	.68
		Europe (n=5)	0.23	0.66 (0.51 to 0.86)		
		North America (n=7)	0.12	0.60 (0.50 to 0.71)		
Publication date	0.60 (0.53 to 0.68), *P* <.001	Intercept: <2015 (n=3)	-0.57	0.57 (0.34 to 0.94)	0.0	.76
		2015-2020 (n=8)	0.13	0.64 (0.53 to 0.78)		
		2020-present (n=4)	0.01	0.57 (0.43 to 0.76)		
Risk of bias	0.60 (0.53 to 0.68), *P* <.001	Intercept: Low (n=6)	-0.58	0.56 (0.42 to 0.74)	0.0	.61
		Medium (n=5)	0.07	0.60 (0.51 to 0.71)		
		Serious (n=4)	0.23	0.71 (0.49 to 1.01)		
Adjustment of HR	0.60 (0.53 to 0.68), *P* <.001	Intercept: Adjusted (n=10)	-0.53	0.59 (0.52 to 0.68)	0.0	.45
		Unadjusted (n=5)	0.14	0.68 (0.49 to 0.94)		
Stage	0.60 (0.53 to 0.68), *P* <.001	Intercept: Stage 0-III (n=1)	-0.56	0.57 (0.40 to 0.83)	0.0	.57
		Stage I (n=1)	0.03	0.59 (0.23 to 1.52)		
		Stage I-III (n=9)	0.19	0.69 (0.55 to 0.87)		
		Stage II-III (n=2)	-0.55	0.33 (0.10 to 1.15)		
		Stage III (n=2)	-0.08	0.53 (0.38 to 0.74)		

Abbreviations: HR, hazard ratio; 95% CI, 95% confidence interval; n, number of studies.

© 2023 de Back T et al. *JAMA Network Open.*

eTable 6. Subgroup analyses for palliative chemotherapy versus no treatment

Meta-analyses	Original values<br>HR (95% CI)	Subgroups<br>Group (n)	Values subgroup analyses<br>HR (95% CI)	Difference between subgroups<br>*P* value
Pall CTx vs no OS	0.48 (0.40 to 0.58), *P*<.001	Asia (n=1)	0.30 (0.17 to 0.53), *P*<.001	.20
Geography		Europe (n=3)	0.45 (0.31 to 0.66), *P*<.001	
		North America (n=4)	0.53 (0.41 to 0.69), *P*<.001	
Pall CTx vs no OS	0.48 (0.40 to 0.58), *P*<.001	<2015 (n=2)	0.36 (0.23 to 0.56), *P*<.001	.40
Publication date		2015-2020 (n=3)	0.46 (0.40 to 0.53), *P*<.001	
		2020-present (n=3)	0.54 (0.37 to 0.79), *P*=.002	
Pall CTx vs no OS	0.48 (0.40 to 0.58), *P*<.001	Low (n=4)	0.50 (0.38 to 0.67), *P*<.001	.29
Risk of bias		Medium (n=3)	0.50 (0.36 to 0.68), *P*<.001	
		Serious (n=1)	0.29 (0.15 to 0.56), *P*<.001	

Abbreviations: HR, hazard ratio; 95% CI, 95% confidence interval; n, number of studies; pall CTx, adjuvant chemotherapy; OS, overall survival.

© 2023 de Back T et al. *JAMA Network Open.*

eTable 7. Meta-regression analysis for palliative chemotherapy versus no chemotherapy overall survival

Variable	Original value<br>HR (95% CI)	Subgroups<br>Group (n)	Regression coefficients<br>Estimates	Predicted HRs<br>HR (95% CI)	Explained heterogeneity<br>I^2 (%)	Test of moderator<br>*P* value
Geography	0.48 (0.40 to 0.58), *P* <.001	Intercept: Asia (n=1)	-1.21	0.30 (0.15 to 0.60)	0.0	.29
		Europe (n=3)	0.41	0.45 (0.31 to 0.65)		
		North America (n=4)	0.57	0.53 (0.41 to 0.69)		
Publication date	0.48 (0.40 to 0.58), *P* <.001	Intercept: <2015 (n=2)	-1.02	0.36 (0.22 to 0.59)	27.6	.27
		2015-2020 (n=3)	0.25	0.46 (0.37 to 0.59)		
		2020-present (n=3)	0.44	0.56 (0.42 to 0.75)		
Risk of bias	0.48 (0.40 to 0.58), *P* <.001	Intercept: Low (n=4)	-0.69	0.50 (0.38 to 0.67)	0.0	.39
		Medium (n=3)	-0.02	0.49 (0.37 to 0.67)		
		Serious (n=1)	-0.55	0.29 (0.14 to 0.61)		
Adjustment of HR	0.48 (0.40 to 0.58), *P* <.001	Intercept: Adjusted (n=7)	-0.70	0.50 (0.42 to 0.60)	22.3	.15
		Unadjusted (n=1)	-0.54	0.29 (0.14 to 0.59)		
Line	0.48 (0.40 to 0.58), *P* <.001	Intercept: 1 (n=2)	-0.65	0.52 (0.33 to 0.83)	0.0	.59
		1-2 (n=2)	-0.36	0.36 (0.21 to 0.64)		
		NS (n=4)	-0.05	0.50 (0.37 to 0.66)		

Abbreviations: HR, hazard ratio; 95% CI, 95% confidence interval; n, number of studies.

© 2023 de Back T et al. *JAMA Network Open.*

eTable 8. Sensitivity analyses for heterogeneous pairwise meta-analyses

	Original values		Adjusted values sensitivity analyses		Excluded studies
Meta-analyses	HR (95% CI)	Heterogeneity	HR (95% CI)	Heterogeneity	Reference
Adjuvant CTx vs no adjuvant CTx OS					
All studies I-III	0.71 (0.60 to 0.84), *P*<.001	I^2 = 63.1%, *P*<.001	0.60 (0.53 to 0.68), *P*<.001	I^2 = 16.9%, *P*=.26	(11), (12), (29)
Stage stratified	0.63 (0.53 to 0.74), *P*<.001	I^2 = 56.3%, *P*=.006	0.58 (0.51 to 0.67), *P*<.001	I^2 = 20.5%, *P*=.24	(11)
D – all studies	0.72 (0.56 to 0.91), *P*=.007	I^2 = 67.3%, *P*=.002	0.67 (0.57 to 0.80), *P*<.001	I^2 = 36.1%, *P*=.15	(29), (36)
D – stage stratified	0.64 (0.50 to 0.82), *P*<.001	I^2 = 61.9%, *P*=.02	0.66 (0.56 to 0.79), *P*<.001	I^2 = 39.1%, *P*=.16	(36)
Palliative CTx vs no palliative CTx OS					
Total cohort	0.44 (0.36 to 0.54), *P*<.001	I^2 = 55.4%, *P*=.02	0.48 (0.40 to 0.58), *P*<.001	I^2 = 43.5%, *P*=.09	(15), (55)
Palliative regimen A vs regimen B OS					
Plat-combo vs other	0.68 (0.41 to 1.12), *P*=.13	I^2 = 74.3%, *P*<.001	0.59 (0.41 to 0.85), *P*=.004	I^2 = 16.4%, *P*=.31	(45), (62), (66)
CTx+bev vs CTx	0.63 (0.37 to 1.07), *P*=.08	I^2 = 59.7%, *P*=.06	0.77 (0.53 to 1.11), *P*=.17	I^2 = 32.0%, *P*=.23	(49)
Palliative regimen A vs regimen B PFS					
F-oxali vs other	0.60 (0.34 to 1.06), *P*=.08	I^2 = 53.1%, *P*=.12	0.46 (0.30 to 0.71), *P*<.001	I^2 = 0.0%, *P*=.85	(66)
Plat-combo vs other	0.70 (0.48 to 1.04), *P*=.07	I^2 = 74.1%, *P*<.001	0.72 (0.54 to 0.95), *P*=.02	I^2 = 39.7%, *P*=.13	(45), (66)
Doublet vs singlet	0.96 (0.68 to 1.37), *P*=.83	I^2 = 51.0%, *P*=.06	0.78 (0.57 to 1.08), *P*=.14	I^2 = 25.9%, *P*=.25	(62), (66)

Abbreviations: HR, hazard ratio; 95% CI, 95% confidence interval; CTx, chemotherapy; vs, versus; OS, overall survival; D, duodenum; Plat-combo, platinum combinations; bev, bevacizumab; PFS, progression-free survival; F-oxali, fluoropyrimidine oxaliplatin.

© 2023 de Back T et al. *JAMA Network Open.*

eTable 9. Sensitivity analyses for heterogeneous pooled medians

	Original values		Adjusted values sensitivity analyses		Excluded studies
Meta-analyses	Months (95% CI)	Heterogeneity	Months (95% CI)	Heterogeneity	Reference
Adjuvant CTx OS					
Adjuvant CTx	41.53 (28.00 to 55.05)	I^2 = 88.1%, *P*<.001	51.91 (45.69 to 58.13)	I^2 = 0.0%, *P*=.47	(18)
No adjuvant CTx	31.29 (24.20 to 38.38)	I^2 = 69.0%, *P*=.04	27.95 (22.96 to 32.95)	I^2 = 0.0%, *P*=.60	(22)
First-line palliative CTx OS					
All CTx	13.98 (12.53 to 15.42)	I^2 = 65.1%, *P*<.001	14.50 (13.48 to 15.51)	I^2 = 0.0%, *P*=.46	(47)
First-line palliative CTx PFS					
All CTx	6.54 (5.62 to 7.45)	I^2 = 73.7%, *P*<.001	6.68 (5.99 to 7.37)	I^2 = 30.4%, *P*=.19	(31), (43), (50)
F-irinotecan	6.32 (3.86 to 8.78)	I^2 = 77.9%, *P*=.003	6.10 (4.66 to 7.55)	I^2 = 0.0%, *P*=.81	(43), (65)
FOLFOX	7.45 (5.93 to 8.97)	I^2 = 56.1%, *P*=.08	8.16 (7.01 to 9.31)	I^2 = 9.2%, *P*=.33	(50)
F-oxaliplatin	6.55 (4.76 to 8.34)	I^2 = 85.5%, *P*<.001	7.49 (6.03 to 8.96)	I^2 = 0.0%, *P*=.57	(43), (50), (65)

Abbreviations: 95% CI, 95% confidence interval; CTx, chemotherapy; OS, overall survival; PFS, progression-free survival; F-irinotecan, fluoropyrimidine irinotecan; FOLFOX, fluorouracil, leucovorin, oxaliplatin; F-oxaliplatin, fluoropyrimidine oxaliplatin.

© 2023 de Back T et al. *JAMA Network Open.*

eTable 10. Certainty of evidence score for meta-analyses with hazard ratios

Meta-analysis	Domains							Effect	Certainty
	n	Study design	Risk of Bias	Inconsistency	Indirectness of evidence	Imprecision of results	Other factors	HR (95% CI)	
eFig 1A. OS benefit of adj CTx vs no CTx for the total subset	15	Observational	M	L	L	L	None	0.60 (0.53 to 0.68)	Moderate
eFig 1B. OS benefit of adj CTx vs no CTx for the stratified subset	12	Observational	M	L	L	L	None	0.58 (0.52 to 0.64)	Moderate
eFig 1C. OS benefit of adj CTx vs no CTx for stage III patients	2	Observational	M	L	L	L	None	0.55 (0.48 to 0.64)	Very low
eFig 2A. RFS benefit of adj CTx vs no CTx in the total subset	8	Observational	M	M	L	L	None	0.92 (0.66 to 1.29)	Low
eFig 2B. RFS benefit of adj CTx vs no CTx in the stratified subset	4	Observational	L	L	L	L	None	0.65 (0.50 to 0.85)	High
eFig 2C. OS benefit of adj CTx vs no CTx for duodenal tumors in the total subset	7	Observational	M	L	L	L	None	0.67 (0.56 to 0.81)	Moderate
eFig 2D. OS benefit of adj CTx vs no CTx for duodenal tumors in the stratified subset	5	Observational	M	L	L	L	None	0.66 (0.54 to 0.80)	Moderate
eFig 3A. OS benefit of adj CTx vs no CTx for jejunal/ileal tumors	3	Observational	M	L	L	L	None	0.70 (0.62 to 0.80)	Moderate
eFig 3B. OS benefit of line-unselected pall CTx vs no CTx	8	Observational	M	L	L	L	None	0.48 (0.40 to 0.58)	Moderate
eFig 3C. OS benefit of first-line pall CTx vs no CTx	2	Observational	L	L	L	L	None	0.50 (0.41 to 0.62)	Moderate
eFig 3D. OS benefit of second-line pall CTx vs no CTx	2	Observational	S	M	L	L	Publication bias	0.40 (0.04 to 4.05)	Very low
eFig 3E. OS benefit of line-unselected pall CTx vs no CTx for patients with duodenal tumors	2	Observational	M	L	L	L	None	0.28 (0.18 to 0.46)	Moderate
eFig 4A. OS benefit of pall CTx for duodenal vs distal tumors	6	Observational	M	L	L	L	None	1.85 (1.43 to 2.38)	Moderate
eFig 4B. OS benefit of pall CTx for duodenal vs jejunal tumors	4	Observational	M	L	L	L	None	1.51 (1.08 to 2.10)	Moderate
eFig 4C. OS benefit of pall CTx for duodenal versus ileal tumors	2	Observational	M	L	L	L	None	1.49 (1.08 to 2.05)	Moderate
eFig 4D. PFS benefit of pall CTx for duodenal vs distal tumors	3	Observational	M	L	L	L	None	1.36 (1.06 to 1.76)	Moderate
eFig 4E. PFS benefit of pall CTx for duodenal vs jejunal tumors	2	Observational	S	L	L	L	None	1.41 (1.04 to 1.90)	Low

Abbreviations: n, number of studies; HR, hazard ratio; 95% CI, 95% confidence interval; eFig, eFigure in the Supplement; OS, overall survival; adj CTx, adjuvant chemotherapy; vs, versus; CTx, chemotherapy; RFS, relapse-free survival; pall CTx, palliative chemotherapy; PFS, progression-free survival.
GRADE classification assessment: L = not serious, M = serious, S = very serious.

© 2023 de Back T et al. *JAMA Network Open.*

eTable 10. Certainty of evidence score for meta-analyses with hazard ratios (continuation)

Meta-analysis	Domains							Effect	
	n	Study design	Risk of Bias	Inconsistency	Indirectness of evidence	Imprecision of results	Other factors	HR (95% CI)	Certainty
eFig 5A. OS benefit of first-line F-oxali vs control	3	Observational	M	L	L	M	None	0.54 (0.30 to 0.99)	Low
eFig 5B. OS benefit of first-line F-cis vs F-mono	2	Observational	M	L	L	L	None	1.68 (0.99 to 2.85)	Moderate
eFig 5C. OS benefit of first-line plat-combo vs control	5	Observational	M	L	L	M	None	0.59 (0.41 to 0.85)	Low
eFig 5D. OS benefit of first-line F-iri vs F-mono	2	Observational	M	L	L	L	None	1.15 (0.62 to 2.11)	Moderate
eFig 5E. OS benefit of first-line doublets vs singlets	6	Observational	M	L	L	M	None	1.09 (0.69 to 1.72)	Low
eFig 6A. OS benefit of first-line CTx+bev vs CTx	3	Observational	M	L	L	L	None	0.77 (0.53 to 1.11)	Moderate
eFig 6B. PFS benefit of first-line F-oxali vs control	2	Observational	M	L	L	M	None	0.46 (0.30 to 0.71)	Low
eFig 6C. PFS benefit of first-line F-cis vs F-mono	2	Observational	M	L	L	L	None	1.55 (0.94 to 2.54)	Moderate
eFig 6D. PFS benefit of first-line plat-combo vs control	7	Observational	M	M	L	M	None	0.72 (0.54 to 0.95)	Very low
eFig 6E. PFS benefit of first-line F-iri vs control	3	Observational	M	M	L	M	None	1.20 (0.75 to 1.93)	Very low
eFig 7A. PFS benefit of first-line doublets vs singlets	5	Observational	M	M	L	M	None	0.78 (0.57 to 1.08)	Very low
eFig 7B. PFS benefit of first-line triplets vs doublets	2	Observational	M	L	L	M	None	1.13 (0.65 to 1.96)	Low
eFig 7C. PFS benefit of first-line CTx+bev vs CTx	2	Observational	M	L	L	M	None	0.62 (0.43 to 0.89)	Low
eFig 7D. OS benefit for first-line F-oxali vs F-mono	2	Observational	M	M	L	L	Publication bias possible	0.66 (0.28 to 1.57)	Very low
eFig 7E. PFS benefit for first-line F-oxali vs F-mono	2	Observational	M	M	L	L	Publication bias possible	0.72 (0.30 to 1.71)	Very low
eFig 7F. PFS benefit for first-line F-iri vs F-mono	2	Observational	M	M	L	L	None	0.94 (0.55 to 1.61)	Low
eFig 8A. CSS benefit for pall CTx vs no CTx	2	Observational	M	L	L	L	None	0.62 (0.49 to 0.79)	Moderate

Abbreviations: n, number of studies; HR: hazard ratio, 95% CI: 95% confidence interval, PFS: progression-free survival, OS: overall survival, F-oxali: fluoropyrimidine-oxaliplatin, F-cis: fluoropyrimidine-cisplatin, F-mono: fluoropyrimidine-monotherapy, Plat-combo: platinum combinations, F-iri: fluoropyrimidine-irinotecan, CTx: chemotherapy, Bev: bevicizumab
GRADE classification assessment: L = not serious, M = serious, S = very serious.

© 2023 de Back T et al. *JAMA Network Open.*

eTable 11. Certainty of evidence score for meta-analyses with medians

Meta-analysis	n	Study design	Risk of Bias	Inconsistency	Indirectness of evidence	Imprecision of results	Other factors	mOS (95% CI)	Certainty
	Domains							Effect	
eFig 8B. Pooled mOS after adj CTx	3	Observational	S	M	L	M	None	51.91 (45.69 to 58.13)	Very low
eFig 8C. Pooled mOS without adj CTx	2	Observational	S	L	L	L	None	27.95 (22.96 to 32.95)	Low
eFig 8D. Pooled mOS after pall CTx	3	Observational	S	L	L	L	None	3.7 (2.71 to 4.68)	Low
eFig 9A. Pooled mOS after pall CTx in the first line	14	Observational	L	L	L	L	None	14.5 (13.48 to 15.51)	High
eFig 9B. Pooled mOS after pall CTx in the second line	4	Observational	M	L	L	L	None	11.44 (9.2 to 13.68)	Moderate
eFig 9C. Pooled mOS after pall CTx in the third line	2	Observational	M	L	L	L	None	8.64 (6.92 to 10.35)	Moderate
eFig 9D. Pooled mOS after pall CTx in the second or third line	6	Observational	S	L	L	M	None	11.12 (9.33 to 12.91)	Very low

Meta-analysis	n	Study design	Risk of Bias	Inconsistency	Indirectness of evidence	Imprecision of results	Other factors	mPFS (95% CI)	Certainty
	Domains							Effect	
eFig 10A. Pooled mPFS after best supportive care	2	Observational	L	L	L	L	None	1.32 (0.88 to 1.77)	Moderate
eFig 10B. Pooled mPFS after pall CTx in the first line	8	Observational	S	L	L	L	None	6.68 (5.99 to 7.37)	Low
eFig 10C. Pooled mPFS after pall CTx in the second line	4	Observational	M	M	L	L	None	3.66 (2.69 to 4.62)	Low
eFig 10D. Pooled mPFS after pall CTx in the second or third line	6	Observational	S	M	L	M	Publication bias	3.10 (2.39 to 3.80)	Very low
eFig 11A. Pooled mPFS after first-line F-oxali	2	Observational	S	L	L	L	None	7.47 (6.03 to 8.96)	Low
eFig 11B. Pooled mPFS after first-line FOLFOX	3	Observational	M	L	L	L	None	8.16 (7.01 to 9.31)	Moderate
eFig 11C. Pooled mPFS after first-line F-iri	2	Observational	M	L	L	L	None	6.10 (4.66 to 7.55)	Moderate
eFig 11D. Pooled mPFS after first-line F-cis	3	Observational	M	M	L	L	None	6.60 (5.44 to 7.77)	Low

Abbreviations: n, number of studies; mOS, median overall survival; 95% CI, 95% confidence interval; adj CTx, adjuvant chemotherapy; pall CTx, palliative chemotherapy; mPFS, median progression-free survival; F-oxali, fluoropyrimidine-oxaliplatin; FOLFOX, fluorouracil, leucovorin, oxaliplatin; F-iri: fluoropyrimidine-irinotecan; F-cis: fluoropyrimidine-cisplatin.
GRADE classification assessment: L = not serious, M = serious, S = very serious.

© 2023 de Back T et al. *JAMA Network Open.*

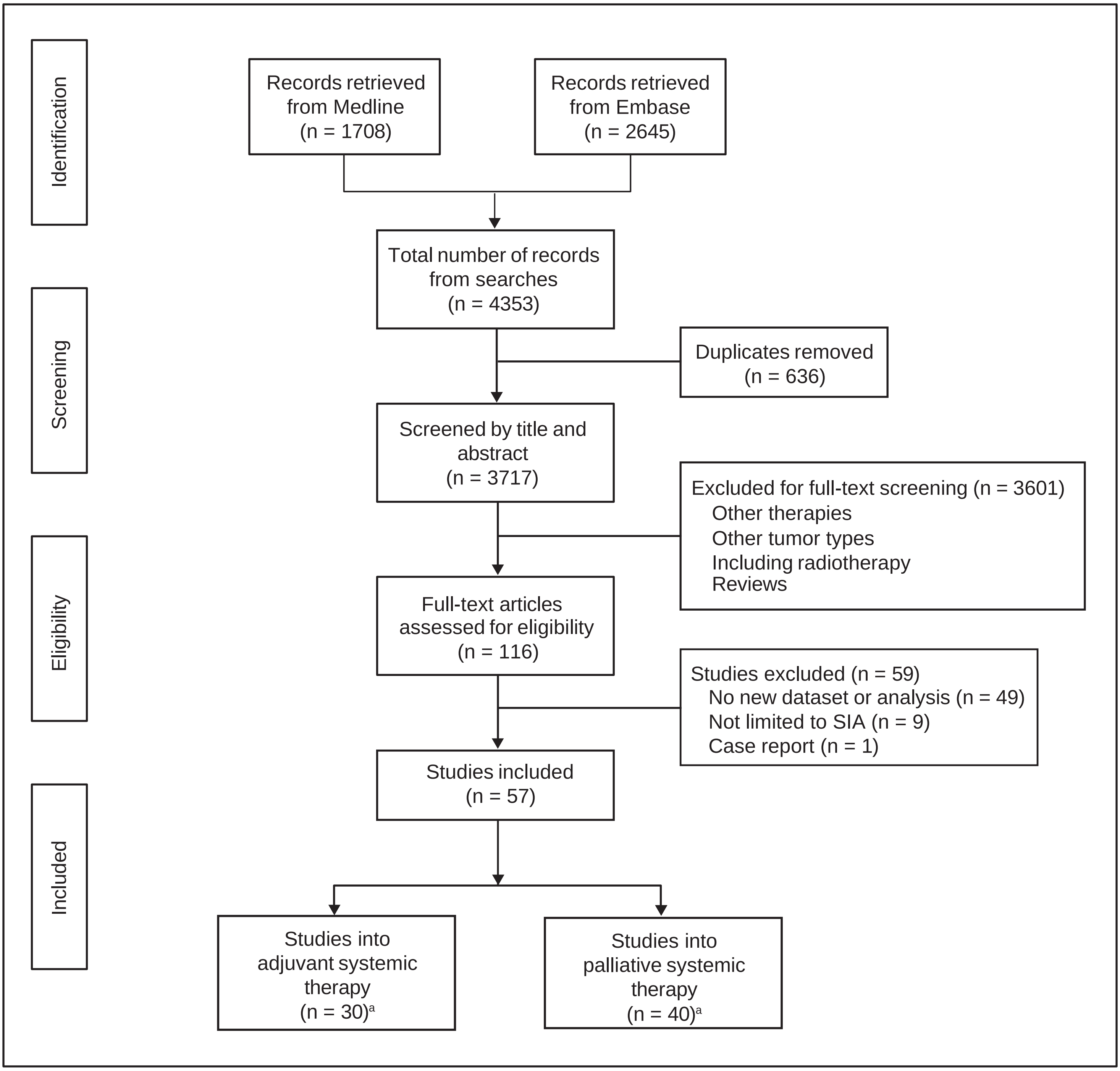

eFigure 1. PRISMA flowchart of study selection.

[a]Thirteen studies described both adjuvant and palliative systemic treatment. SIA, small intestinal adenocarcinoma.

© 2023 de Back T et al. *JAMA Network Open.*

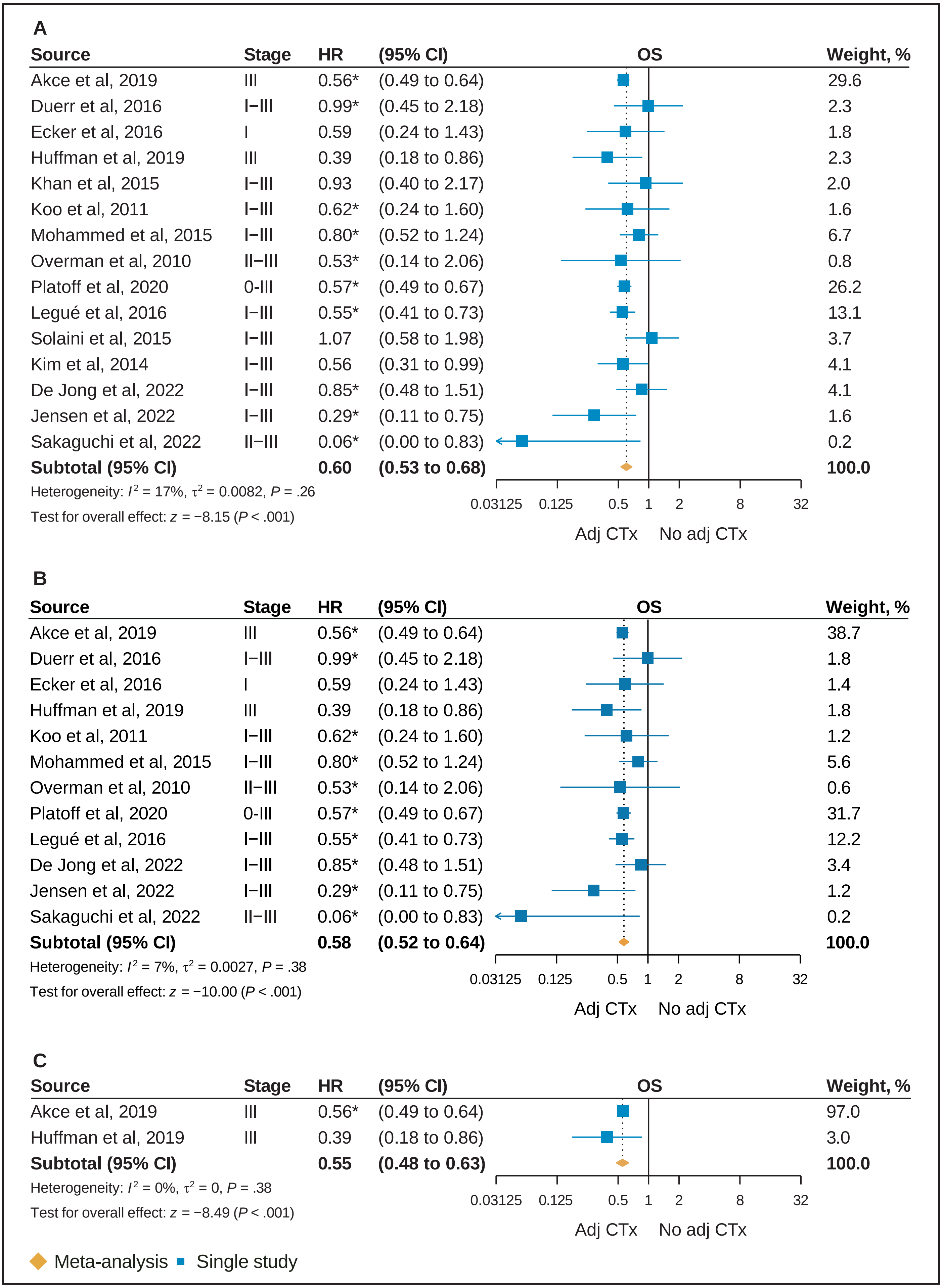

eFigure 2. Forest plots.

Overall survival benefit of adjuvant chemotherapy for the total cohort (A), for the stage-stratified cohort (B), and for the cohort of stage III patients (C). *Hazard ratios adjusted for stage, age, differentiation and nodal involvement. HR, hazard ratio; 95% CI, 95% confidence interval; OS, overall survival; Adj CTx, adjuvant chemotherapy.

© 2023 de Back T et al. *JAMA Network Open.*

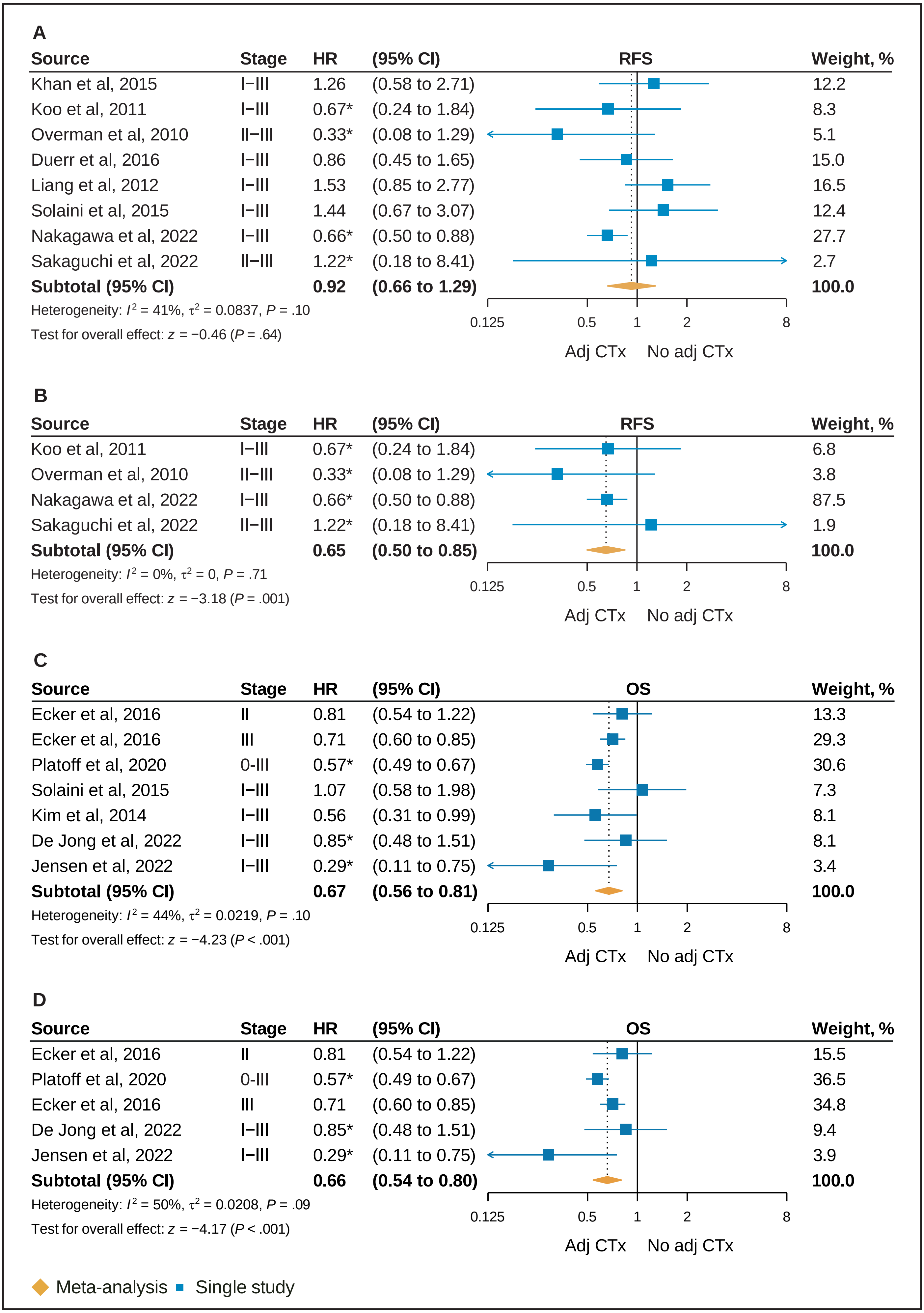

eFigure 3. Forest plots.

Relapse-free survival benefit of adjuvant chemotherapy in the total cohort (A), and in the stage-stratified cohort (B), overall survival benefit of adjuvant chemotherapy for duodenal adenocarcinomas in the total cohort (C), and in the stage-stratified cohort (D). *Hazard ratios adjusted for stage, nodal involvement, differentiation and resection margin. HR, hazard ratio; 95% CI, 95% confidence interval; RFS, relapse-free survival; Adj CTx, adjuvant chemotherapy; OS, overall survival.

© 2023 de Back T et al. *JAMA Network Open.*

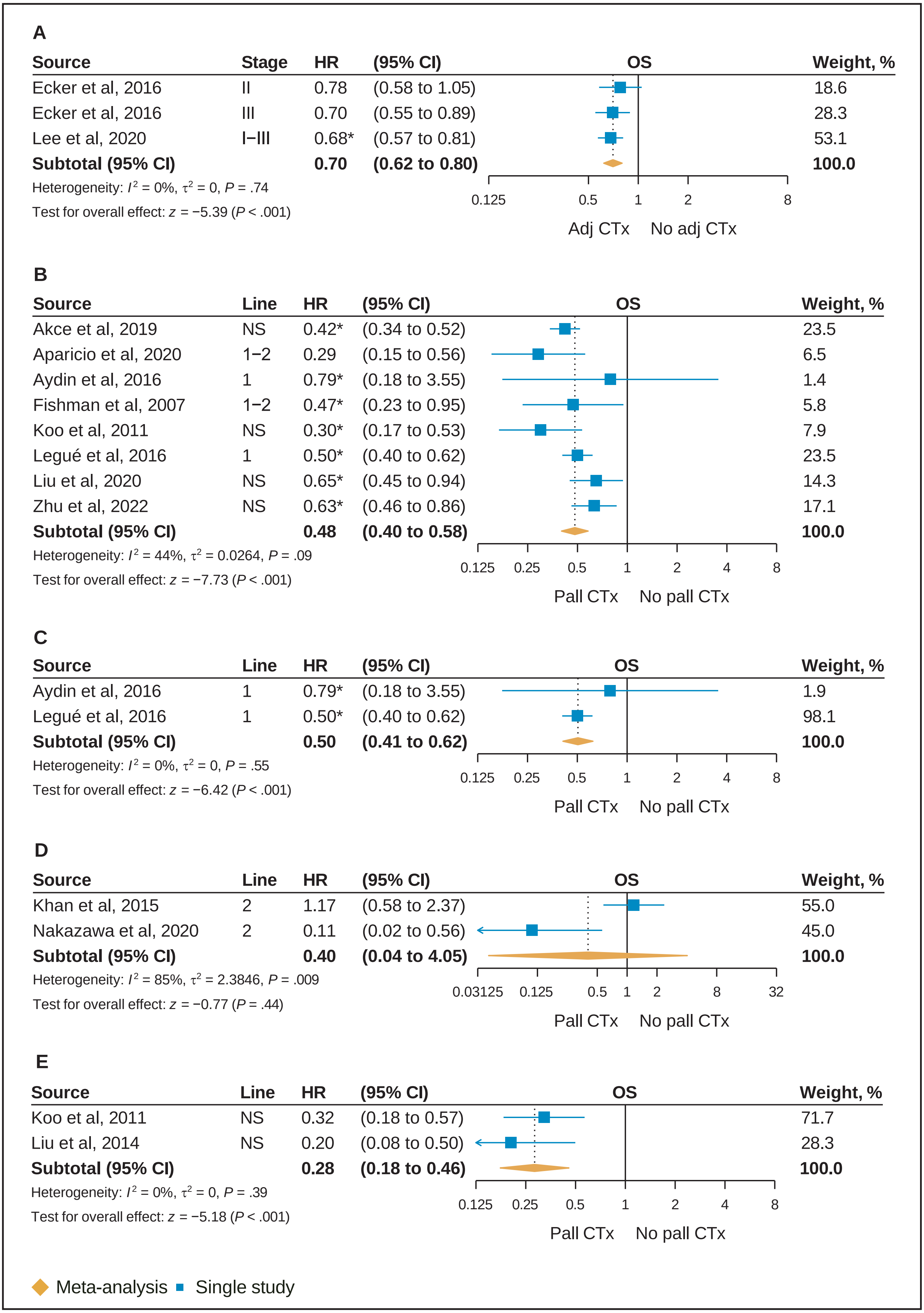

eFigure 4. Forest plots.

Overall survival benefit of adjuvant chemotherapy for jejunal/ileal adenocarcinomas (A), overall survival benefit of line-unselected palliative chemotherapy (B), of first-line palliative chemotherapy (C), of second-line palliative chemotherapy (D), and of line-unselected palliative chemotherapy for patients with duodenal adenocarcinomas (E). *Hazard ratios adjusted for age, sex, localization and surgery. HR, hazard ratio; 95% CI, 95% confidence interval; OS, overall survival; Adj CTx, adjuvant chemotherapy; NS, not specified; Pall CTx, palliative chemotherapy.

© 2023 de Back T et al. *JAMA Network Open.*

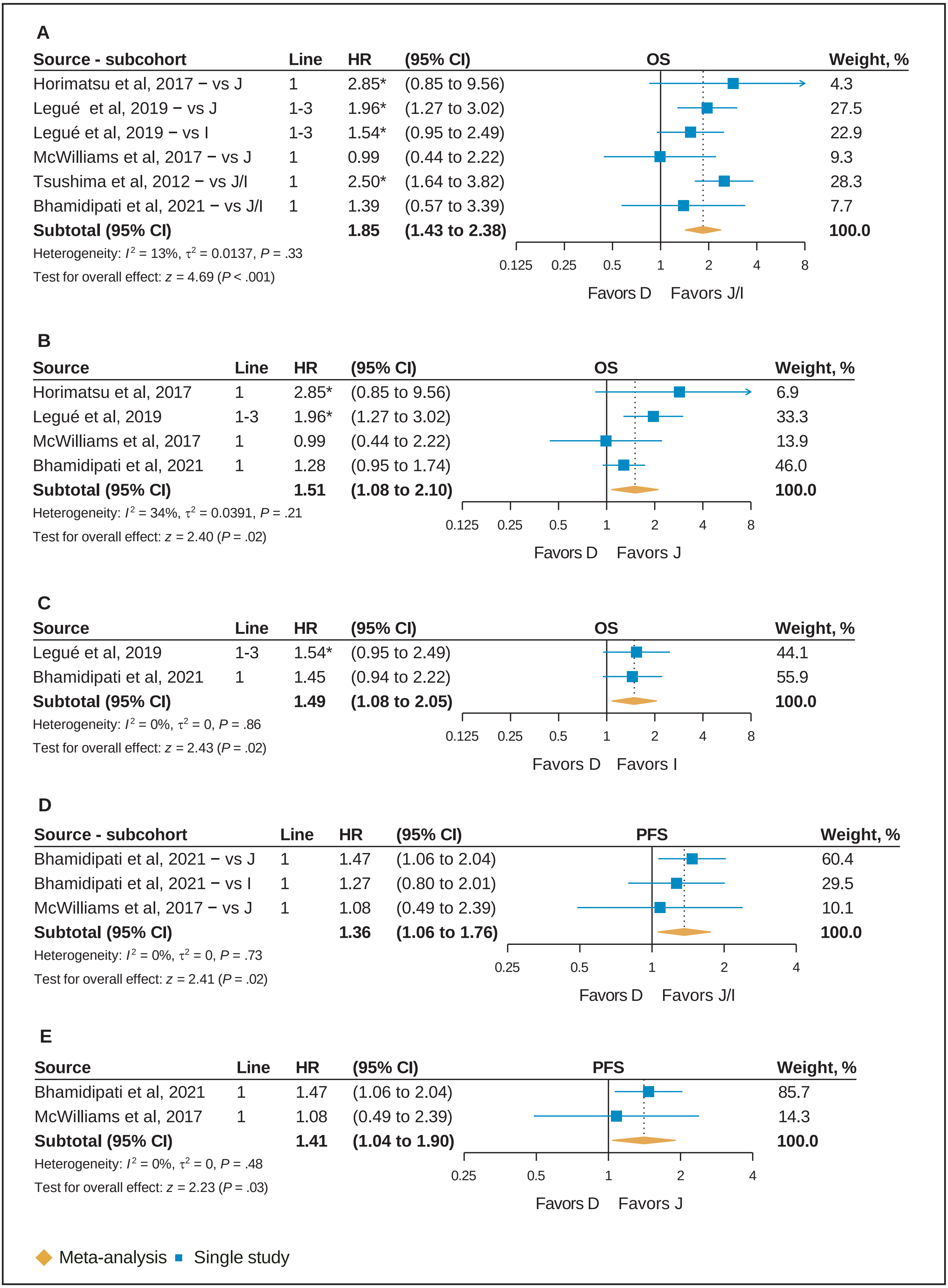

eFigure 5. Forest plots.

Overall survival benefit of palliative chemotherapy for duodenal versus distal adenocarcinomas (A), versus jejunal adenocarcinomas (B), and versus ileal adenocarcinomas (C), progression-free survival benefit of palliative chemotherapy for duodenal versus distal adenocarcinomas (D), and versus jejunal adenocarcinomas (E). *Hazard ratios adjusted for performance score, CEA level and metastatic sites. HR, hazard ratio; 95% CI, 95% confidence interval; OS, overall survival; J, jejunum; I, ileum; D, duodenum; PFS, progression-free survival.

© 2023 de Back T et al. *JAMA Network Open.*

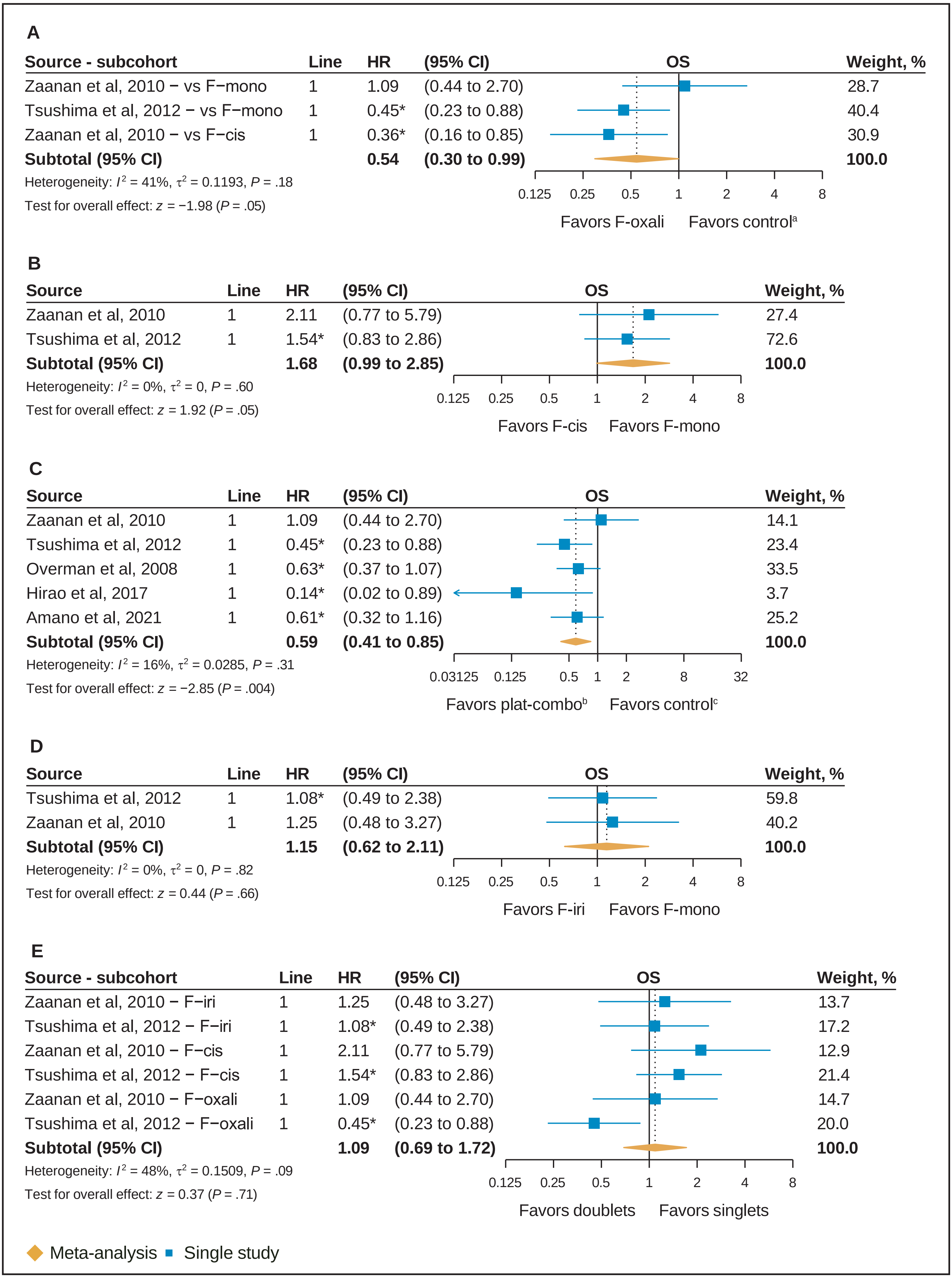

eFigure 6. Forest plots.

Overall survival benefit of first-line F-oxali vs control (A), F-cis vs F-mono (B), plat-combo versus control (C), F-iri vs F-mono (D), and doublets vs singlets (E). *Hazard ratios adjusted for surgery, performance score and CEA level. [a]F-mono, F-cis. [b]Combinations with oxaliplatin, carboplatin and cisplatin. [c]F-mono, plat-mono, non-plat combo, non-plat/ non-F. HR, hazard ratio; 95% CI, 95% confidence interval; OS, overall survival; F-mono, fluoropyrimidine-monotherapy; F-cis, fluoropyrimidine- cisplatin; F-oxali, fluoropyrimidine-oxaliplatin; Plat-combo, platinum combinations; F-iri, fluoropyrimidine-irinotecan.

© 2023 de Back T et al. *JAMA Network Open.*

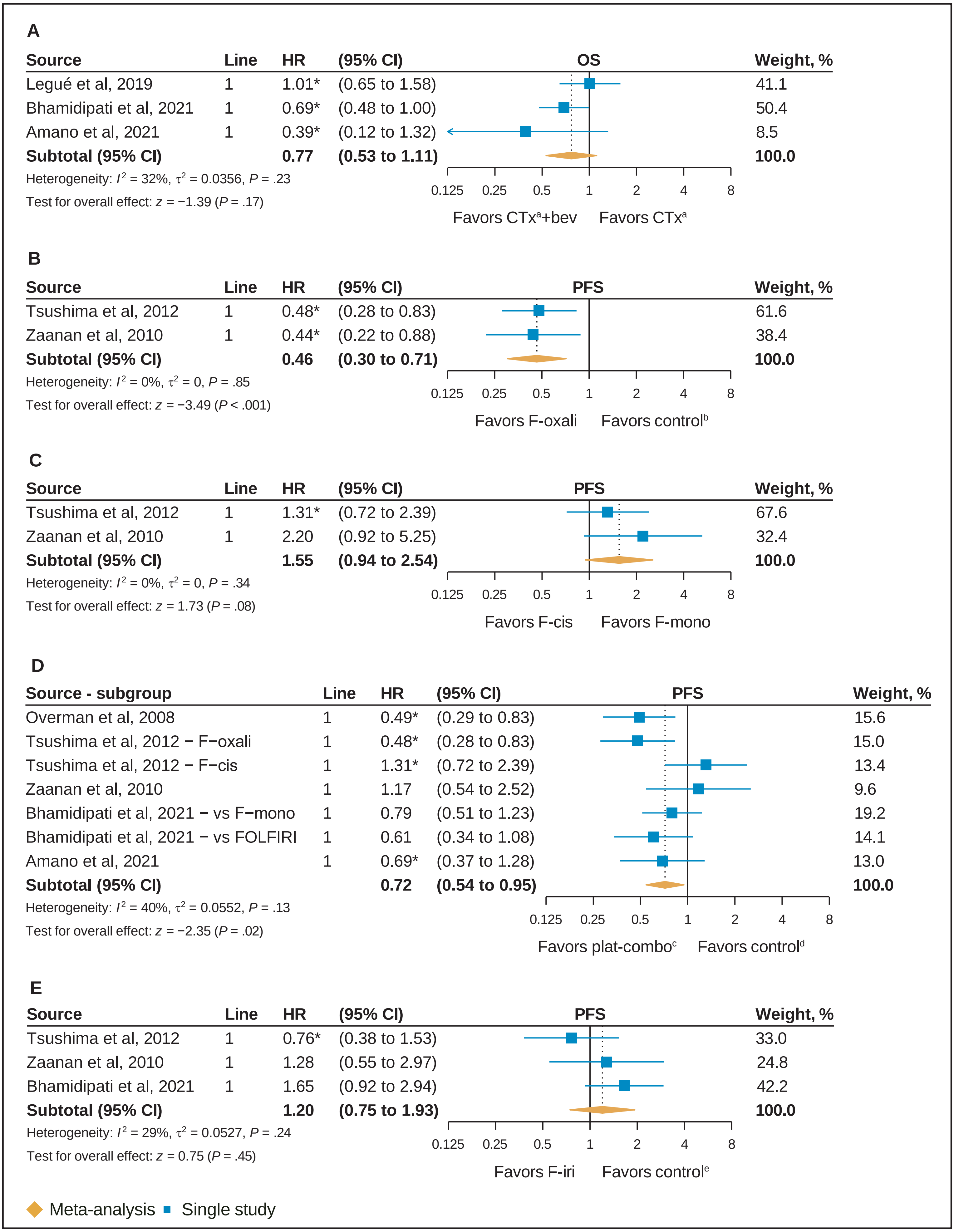

eFigure 7. Forest plots.

Overall survival benefit of CTx+bev vs CTx (A), progression-free survival benefit of F-oxali vs control (B), F-cis vs F-mono (C), plat-combo vs control (D), and F-iri vs control (E). *Hazard ratios adjusted for surgery, localization, CEA level and performance score. [a]Plat-combo, F-mono, F-iri, F-irinox. [b]F-mono and F-cis. [c]Combinations with oxaliplatin, carboplatin and cisplatin. [d]F-mono, plat-mono, non-plat combo, non-plat/non-F, FOLFIRI. [e]F-mono and plat-combo. HR, hazard ratio; 95% CI, 95% confidence interval; OS, overall survival; CTx, chemotherapy; Bev, bevacizumab; PFS, progression-free survival; F-oxali, fluoropyrimidine-oxaliplatin; F-cis, fluoropyrimidine- cisplatin; F-mono, fluoropyrimidine-monotherapy; Plat-combo, platinum combinations; F-iri, fluoropyrimidine- irinotecan.

© 2023 de Back T et al. *JAMA Network Open.*

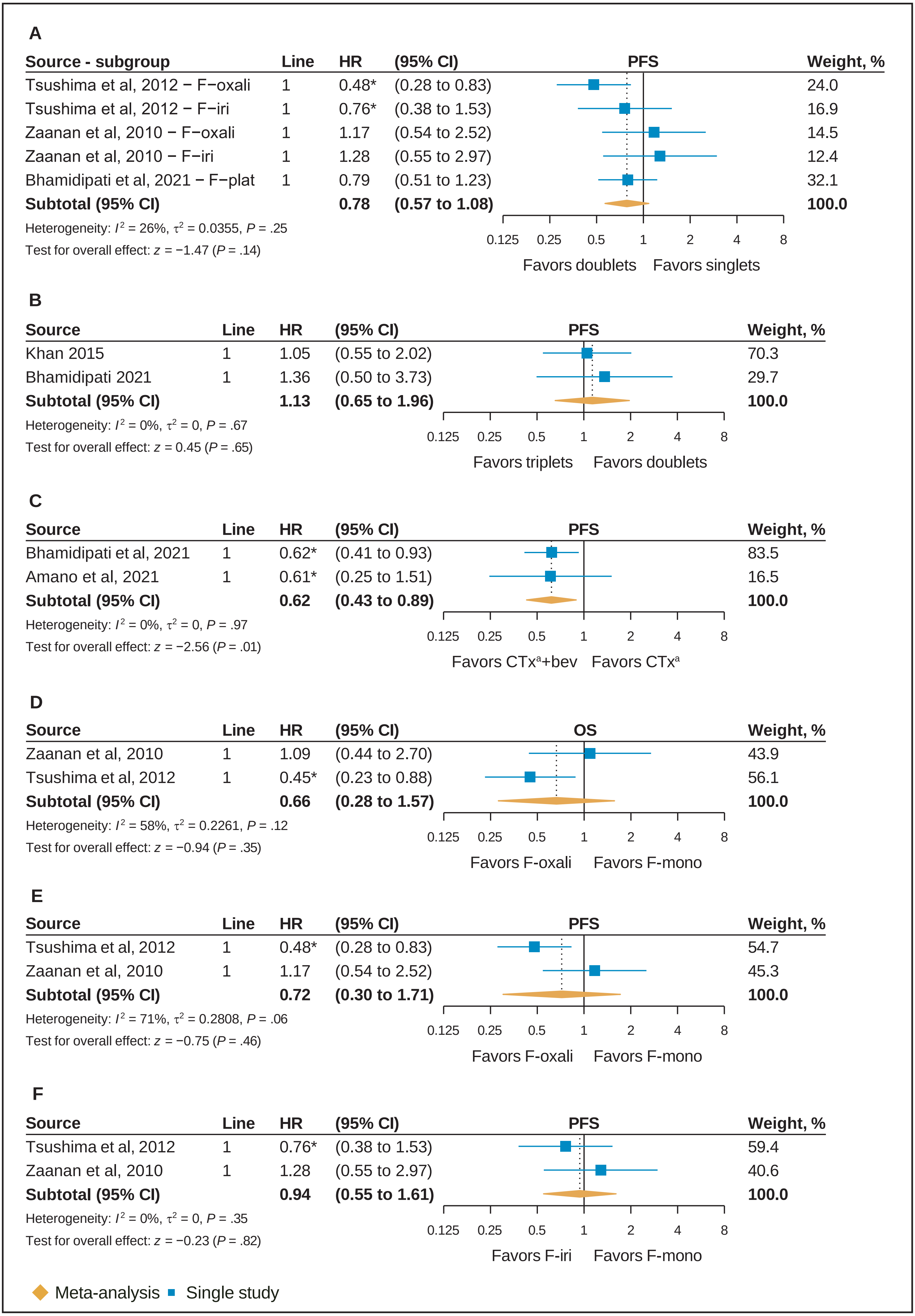

© 2023 de Back T et al. *JAMA Network Open.*

eFigure 8. Forest plots (previous page).

Progression-free survival benefit of doublets vs singlets (A), triplets vs doublets (B), and CTX+bev vs CTx (C), overall survival benefit for F-oxali vs F-mono (D), progression-free survival benefit for F-oxali vs F-mono (E), and F-iri vs F-mono (F). *Hazard ratios adjusted for surgery, performance score and histology. [a]Plat-combo, F-mono, F-iri, F-irinox. HR, hazard ratio; 95% CI, 95% confidence interval; PFS, progression-free survival; F-oxali, fluoropyrimidine-oxaliplatin; F-iri, fluoropyrimidine-irinotecan; F-plat, fluoropyrimidine-platinum; CTx, chemotherapy; Bev, bevacizumab; OS, overall survival; F-mono, fluoropyrimidine-monotherapy.

A

Source	Line	HR	(95% CI)	Weight, %
Liu et al, 2020	NS	0.65*	(0.45 to 0.94)	42.1
Zhu et al, 2022	NS	0.61*	(0.44 to 0.83)	57.9
**Subtotal (95% CI)**		**0.62**	**(0.49 to 0.79)**	**100.0**

Heterogeneity: I^2 = 0%, τ^2 = 0, P = .78

Test for overall effect: z = −3.83 (P < .001)

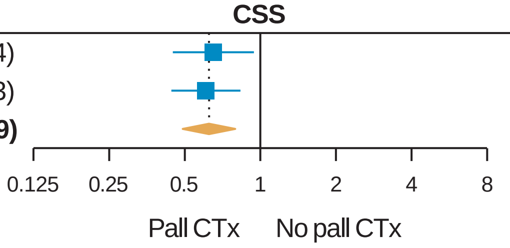

B

Source	Stage	Median	(95% CI)	Weight, %
Guo et al, 2014	I-III	46.00	(18.82 to 73.18)	5.2
Huffman et al, 2019	III	33.80	(1.70 to 65.90)	3.8
Kaslow et al, 2022	I-III	53.00	(46.48 to 59.52)	91.0
**Subtotal (95% CI)**		**51.91**	**(45.69 to 58.13)**	**100.0**

Heterogeneity: I^2 = 0%, τ^2 = 0, P = .47

Test for overall effect: z = 16.36 (P < .001)

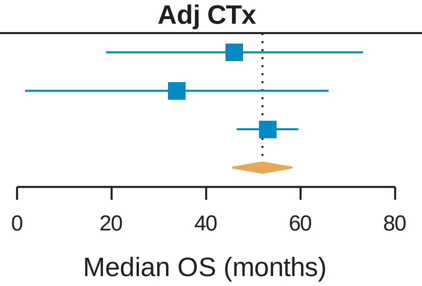

C

Source	Stage	Median	(95% CI)	Weight, %
Fishman et al, 2006	III	28.50	(23.10 to 33.90)	85.6
Huffman et al, 2019	I-III	24.70	(11.55 to 37.85)	14.4
**Subtotal (95% CI)**		**27.95**	**(22.96 to 32.95)**	**100.0**

Heterogeneity: I^2 = 0%, τ^2 = 0, P = .60

Test for overall effect: z = 10.97 (P < .001)

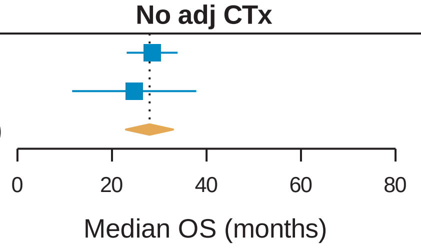

D

Source	Line	Median	(95% CI)	Weight, %
Aparicio et al, 2020	1	2.20	(0.00 to 4.79)	13.5
Koo et al, 2011	1	4.10	(3.10 to 5.10)	68.1
Nakazawa et al, 2020	2	3.30	(1.10 to 5.50)	18.4
**Subtotal (95% CI)**		**3.70**	**(2.71 to 4.68)**	**100.0**

Heterogeneity: I^2 = 0%, τ^2 = 0.1099, P = .37

Test for overall effect: z = 7.36 (P < .001)

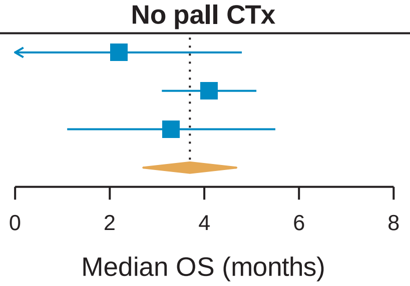

◆ Meta-analysis ■ Single study

eFigure 9. Forest plots.

Cancer-specific survival benefit for palliative chemotherapy versus no chemotherapy (A), pooled median overall survival after adjuvant chemotherapy (B), without adjuvant chemotherapy (C), and after palliative chemotherapy (D). *Hazard ratios adjusted for age, localization, TNM-stage and differentiation. HR, hazard ratio; 95% CI, 95% confidence interval; CSS, cancer-specific survival; NS, not specified; pall CTx, palliative chemotherapy; adj CTx, adjuvant chemotherapy; OS, overall survival.

© 2023 de Back T et al. *JAMA Network Open.*

A

Source - subgroup	Line	Median	(95% CI)	Weight, %
Hirao et al, 2017	1	14.80	(0.00 to 36.58)	0.2
Overman et al, 2008	1	13.00	(9.92 to 16.08)	9.3
Xiang et al, 2012	1	15.20	(11.00 to 19.40)	5.4
Zaanan et al, 2010	1	15.10	(11.39 to 18.81)	6.8
Bhamidipati et al, 2021	1	16.30	(13.45 to 19.15)	10.6
Aydin et al, 2016 − mFOLFOX6	1	13.00	(10.45 to 15.55)	12.8
Aydin et al, 2016 − FOLFIRI	1	16.00	(9.48 to 22.52)	2.3
Aydin et al, 2016 − F−cis	1	15.00	(13.42 to 16.58)	25.4
Aydin et al, 2016 − Gemcitabin	1	11.00	(2.94 to 19.06)	1.6
Horimatsu et al, 2017	1	17.30	(13.16 to 21.44)	5.5
McWilliams et al, 2017	1	13.40	(9.49 to 17.31)	6.1
Yhim et al, 2015	1	11.30	(8.10 to 14.50)	8.8
Amano et al, 2021 − I	1	17.50	(12.00 to 23.00)	3.2
Amano et al, 2021 − D/J	1	17.00	(9.72 to 24.28)	1.9
**Subtotal (95% CI)**		**14.50**	**(13.48 to 15.51)**	**100.0**

Heterogeneity: I^2 = 0%, τ^2 = 0.4074, *P* = .46

Test for overall effect: *z* = 27.96 (*P* < .001)

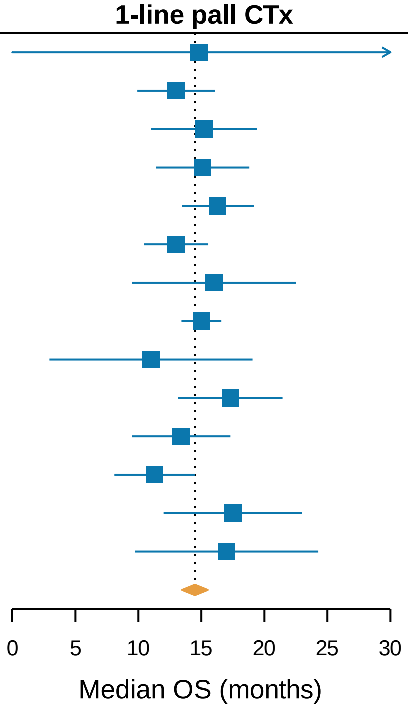

B

Source	Line	Median	(95% CI)	Weight, %
Fishman et al, 2007	2	9.40	(6.70 to 12.10)	33.7
Nakazawa et al, 2020	2	15.60	(7.54 to 23.66)	6.9
Zaanan et al, 2011	2	10.50	(5.11 to 15.89)	13.7
Bhamidipati et al, 2021	2	12.60	(10.76 to 14.44)	45.7
**Subtotal (95% CI)**		**11.44**	**(9.20 to 13.68)**	**100.0**

Heterogeneity: I^2 = 37%, τ^2 = 1.9703, *P* = .19

Test for overall effect: *z* = 10.02 (*P* < .001)

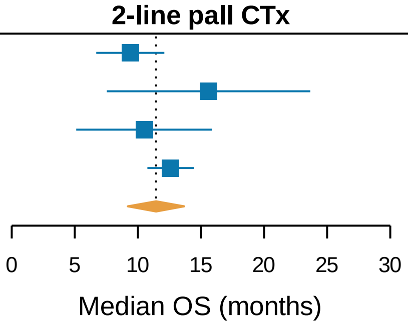

C

Source	Line	Median	(95% CI)	Weight, %
Fishman et al, 2007	3	7.20	(4.00 to 10.40)	27.1
Bhamidipati et al, 2021	3	9.17	(7.32 to 11.02)	72.9
**Subtotal (95% CI)**		**8.64**	**(6.92 to 10.35)**	**100.0**

Heterogeneity: I^2 = 8%, τ^2 = 0.1636, *P* = .30

Test for overall effect: *z* = 9.86 (*P* < .001)

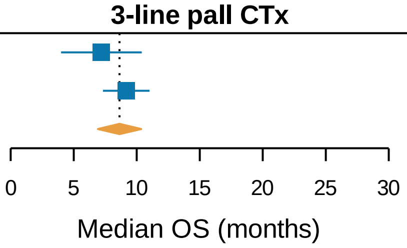

D

Source	Line	Median	(95% CI)	Weight, %
Fishman et al, 2007	2	9.40	(6.70 to 12.10)	26.2
Nakazawa et al, 2020	2	15.60	(7.54 to 23.66)	4.6
Overman et al, 2018	2/3	10.90	(7.00 to 14.80)	15.9
Pedersen et al, 2019	2/3	7.10	(0.00 to 14.31)	5.6
Zaanan et al, 2018	2	10.50	(5.11 to 15.89)	9.4
Bhamidipati et al, 2021	2	12.60	(10.76 to 14.44)	38.3
**Subtotal (95% CI)**		**11.12**	**(9.33 to 12.91)**	**100.0**

Heterogeneity: I^2 = 21%, τ^2 = 1.2908, *P* = .28

Test for overall effect: *z* = 12.18 (*P* < .001)

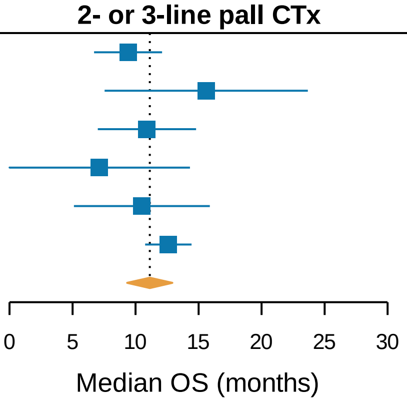

◆ Meta-analysis ■ Single study

© 2023 de Back T et al. *JAMA Network Open.*

eFigure 10. Forest plots (previous page).
Median overall survival after palliative chemotherapy in the first line (A), second line (B), third line (C), and second or third line (D). 95% CI, 95% confidence interval; pall CTx, palliative chemotherapy; F-cis, fluoropyrimidine-cisplatin; I, ileum; D, duodenum; J, jejunum; OS, overall survival.

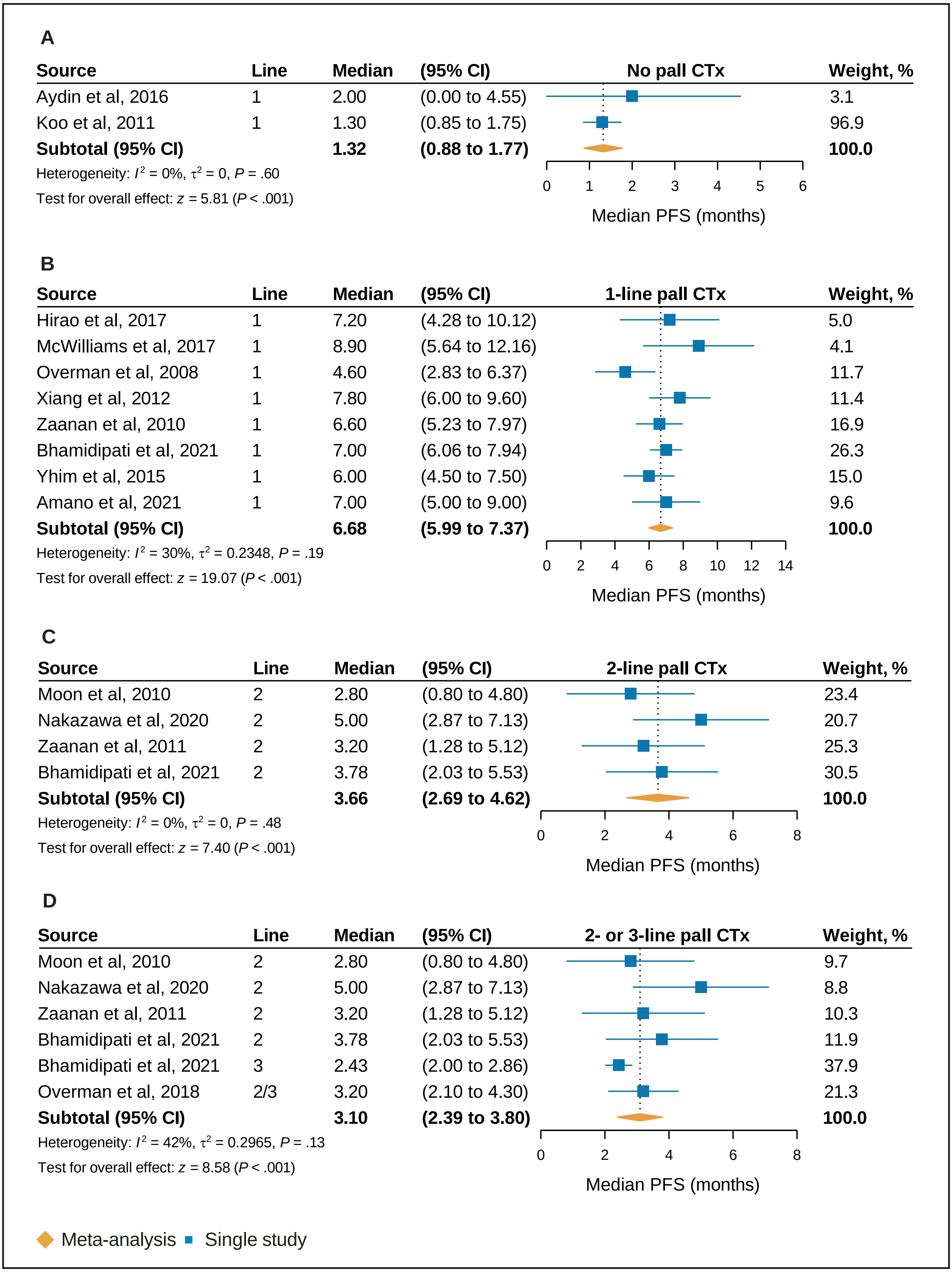

eFigure 11. Forest plots.
Median progression-free survival after best supportive care (A), after palliative chemotherapy in the first line (B), second line (C), and second or third line (D). 95% CI, 95% confidence interval; pall CTx, palliative chemotherapy; PFS, progression-free survival.

© 2023 de Back T et al. *JAMA Network Open.*

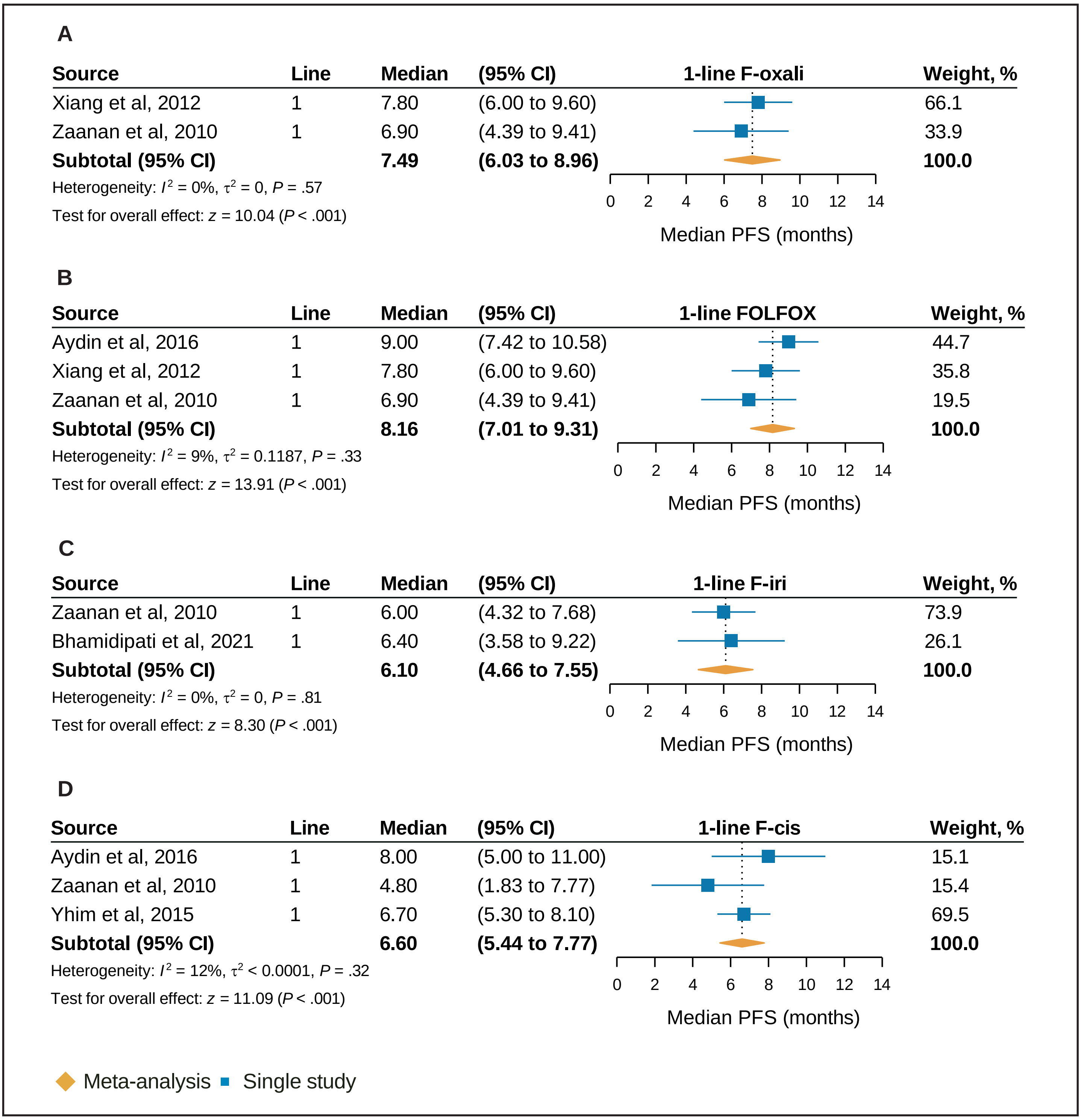

eFigure 12. Forest plots.

Median progression-free survival after first-line F-oxali (A), first-line FOLFOX (B), first-line F-iri (C) and first-line F-cis (D). 95% CI, 95% confidence interval; F-oxali, fluoropyrimidine-oxaliplatin; PFS, progression-free survival; F-iri, fluoropyrimidine-irinotecan; F-cis, fluoropyrimidine-cisplatin.

© 2023 de Back T et al. *JAMA Network Open.*

A*

Fluoropyrimidine-cisplatin	0.67 (0.22 to 2.03), *P*=.48	0.59 (0.27 to 1.25), *P*=.18	0.38 (0.13 to 1.14), *P*=.08
	Fluoropyrimidine-irinotecan	0.87 (0.39 to 1.97), *P*=.74	0.56 (0.19 to 1.75), *P*=.31
		Fluoropyrimidine monotherapy	0.64 (0.30 to 1.44), *P*=.26
			Fluoropyrimidine-oxaliplatin

B*

Fluoropyrimidine-cisplatin	0.59 (0.19 to 1.88), *P*=.37	0.62 (0.27 to 1.35), *P*=.24	0.42 (0.14 to 1.29), *P*=.13
	Fluoropyrimidine-irinotecan	1.05 (0.46 to 2.33), *P*=.90	0.73 (0.24 to 2.19), *P*=.57
		Fluoropyrimidine monotherapy	0.68 (0.32 to 1.54), *P*=.34
			Fluoropyrimidine-oxaliplatin

eFigure 13. Network meta-analysis for first-line chemotherapy regimens.

Each cell displays the hazard ratio, 95% confidence interval and *P* value for the column-defining intervention relative to the row-defining intervention. Comparative effect of first-line regimens on overall survival (A), and progression-free survival (B). *Studies included in network meta-analysis: Tsushima et al,[62] 2012 and Zaanan et al,[66] 2010.

© 2023 de Back T et al. *JAMA Network Open.*

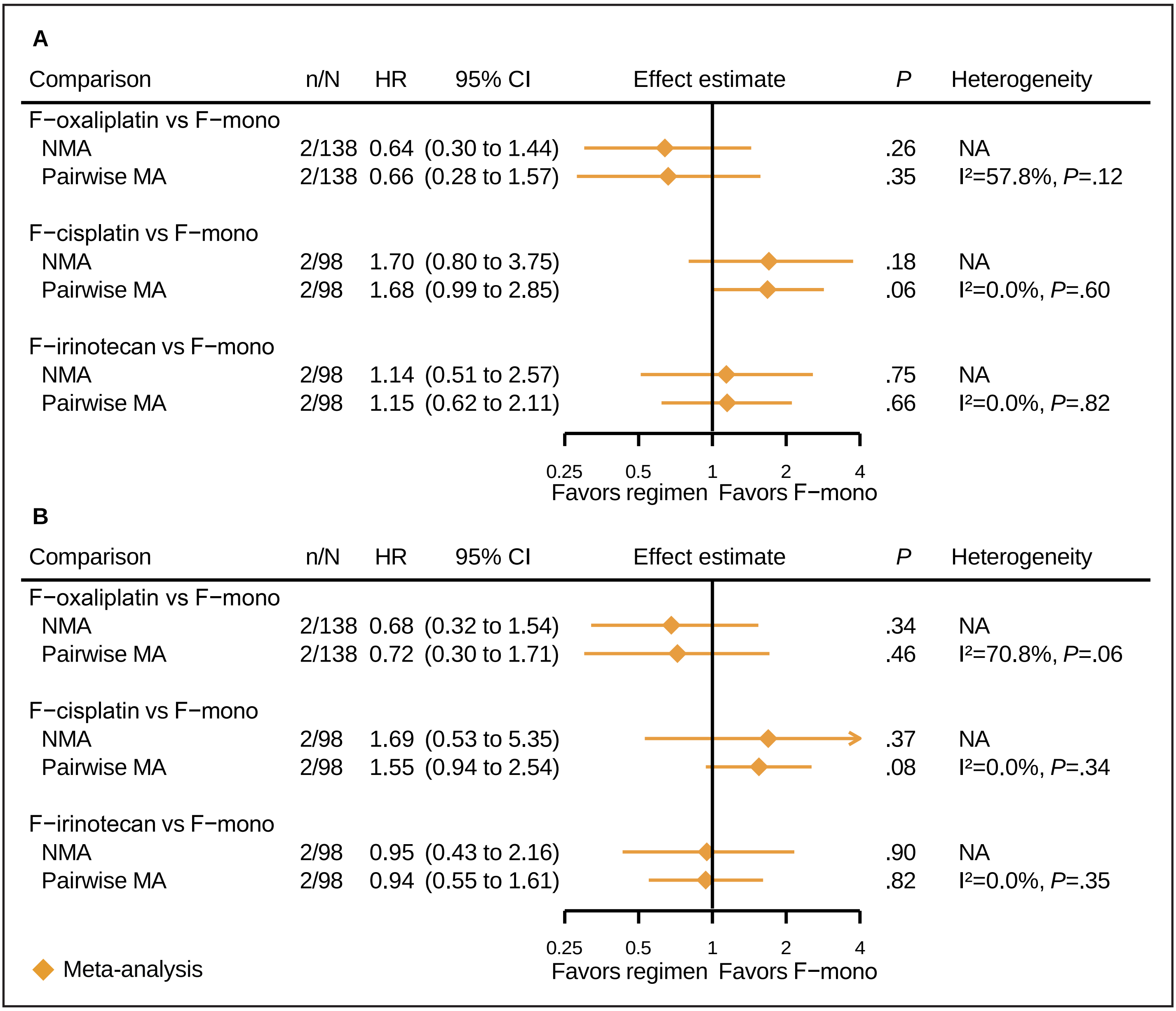

eFigure 14. Comparison of pairwise and network meta-analyses in the first-line setting.
Comparison between pairwise and network meta-analyses for overall survival (A) and progression-free survival (B). n/N, number of studies/number of patients; HR, hazard ratio; 95% CI, 95% confidence interval; *P*, *P* value; F-oxali, fluoropyrimidine-oxaliplatin; F-mono, fluoropyrimidine monotherapy; F-cis, fluoropyrimidine-cisplatin; F-iri, fluoropyrimidine-irinotecan; NMA, network meta-analysis; MA, meta-analysis; NA, not applicable.

© 2023 de Back T et al. *JAMA Network Open.*

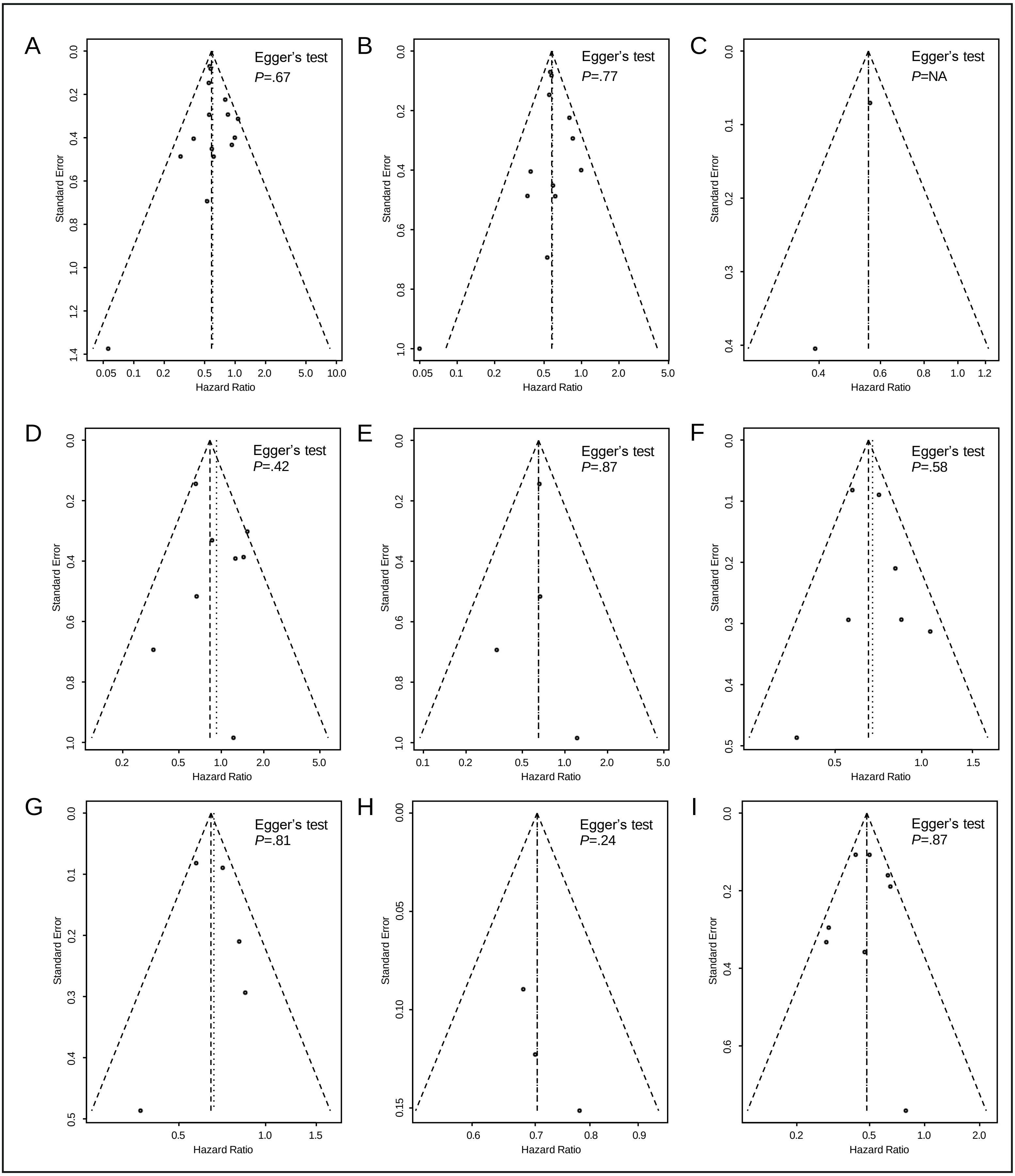

eFigure 15. Evaluation of publication bias.

Funnel plots, including Egger's test *P* values, for adj CTx total cohort OS (A), adj CTx stage stratified OS (B), adj CTx stage III OS (C), adj CTx all studies RFS (D), adj CTx stratified studies RFS (E), adj CTx duodenum all studies OS (F), adj CTx duodenum stratified OS (G), adj CTx jejunum and ileum stratified OS (H), pall CTx total cohort OS (I). Adj CTx, adjuvant chemotherapy; OS, overall survival; RFS, relapse-free survival; pall CTx, palliative chemotherapy.

© 2023 de Back T et al. *JAMA Network Open.*

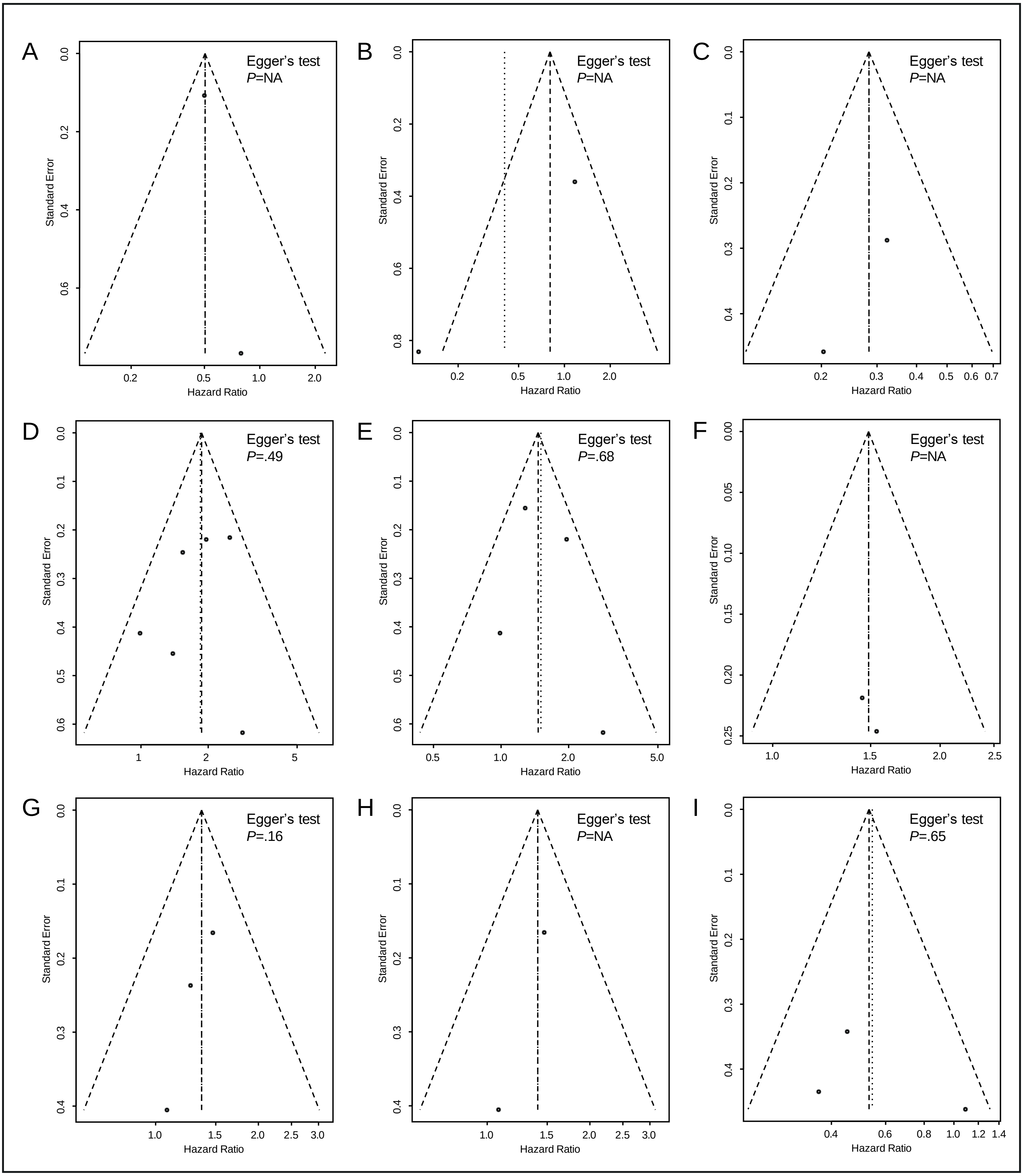

eFigure 16. Evaluation of publication bias.

Funnel plots, including Egger's test *P* values, for pall CTx first line OS (A), pall CTx second line OS (B), pall CTx duodenum OS (C), pall CTx D vs J/I OS (D), pall CTx D vs J OS (E), pall CTx D vs I OS (F), pall CTx D vs J/I PFS (G), pall CTx D vs J PFS (H), pall CTx F-oxali vs other OS (I). Pall CTx, palliative chemotherapy; OS, overall survival; D, duodenum; J/I; jejunum/ileum; PFS, progression-free survival; F-oxali, fluoropyrimidine-oxaliplatin.

© 2023 de Back T et al. *JAMA Network Open.*

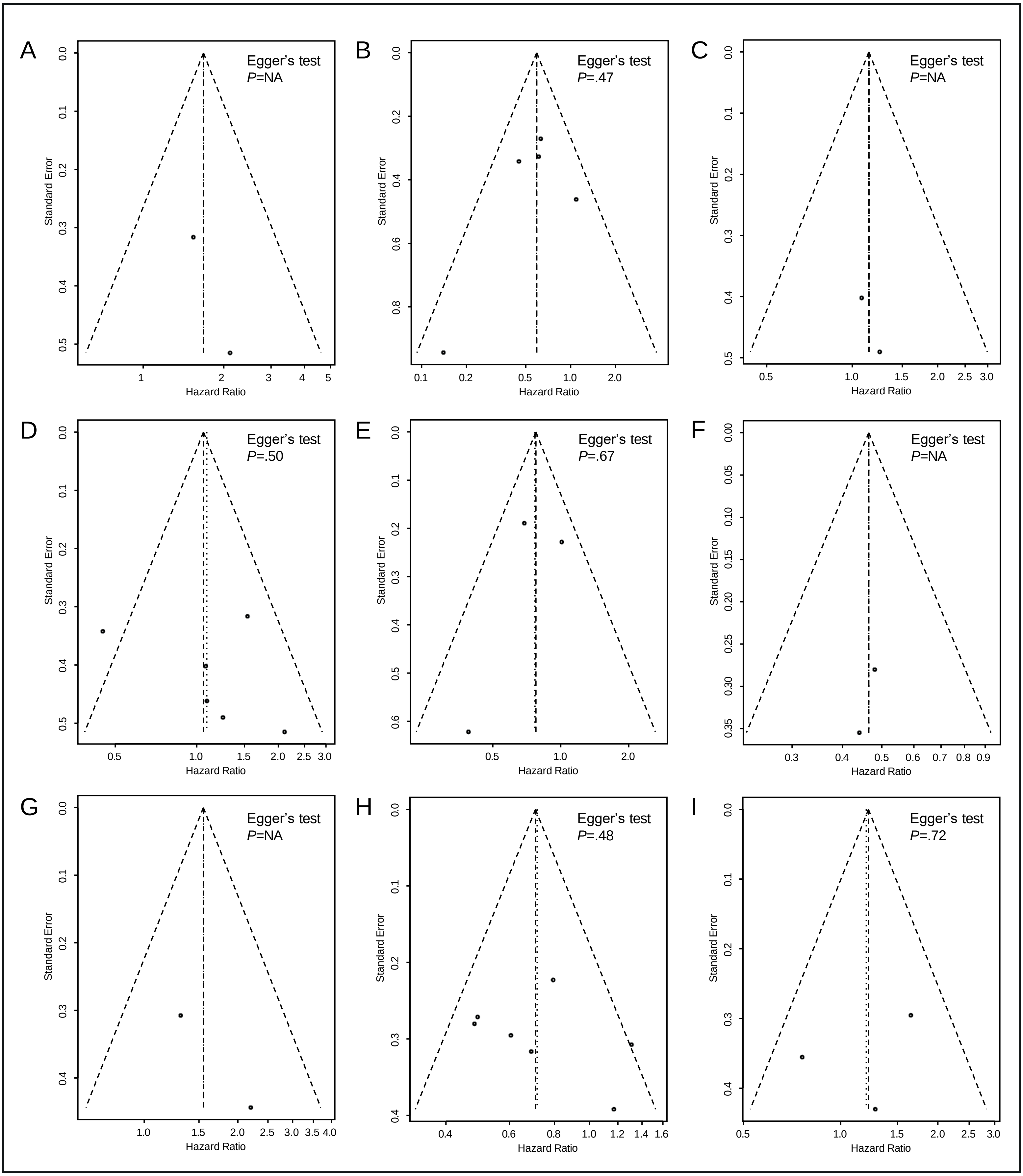

eFigure 17. Evaluation of publication bias.

Funnel plots, including Egger's test *P* values, for pall CTx, F-cis versus F-mono OS (A), plat-combo versus other OS (B), F-iri versus F-mono OS (C), doublet versus singlet OS (D), CTx+bev versus CTx OS (E), F-oxali versus other PFS (F), F-cis versus F-mono PFS (G), plat-combo versus other PFS (H), F-iri versus other PFS (I). Pall CTx, palliative chemotherapy; F-cis, fluoropyrimidine-cisplatin; F-mono, fluoropyrimidine monotherapy; Plat-combo, platinum combinations; F-iri, fluoropyrimidine-irinotecan; CTx+bev, chemotherapy with bevacizumab; CTx, chemotherapy; F-oxali, fluoropyrimidine-oxaliplatin; PFS, progression-free survival.

© 2023 de Back T et al. *JAMA Network Open.*

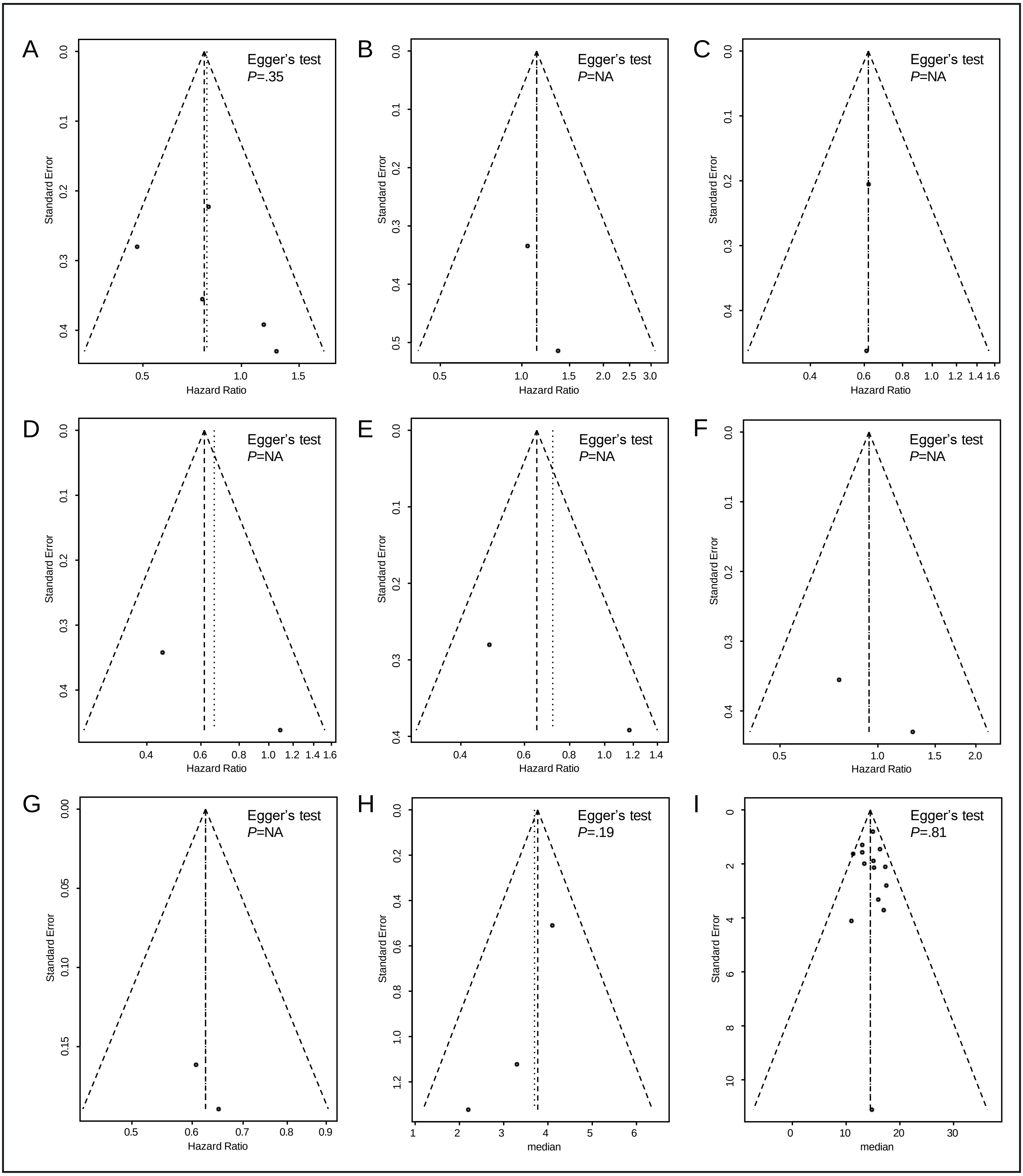

eFigure 18. Evaluation of publication bias.

Funnel plots, including Egger's test *P* values, for pall CTx, doublet versus singlet PFS (A), triplet versus doublet PFS (B), CTx+bev versus CTx PFS (C), F-oxali versus F-mono OS (D), F-oxali versus F-mono PFS (E), F-iri versus F-mono PFS (F), CTx versus no CTx CSS (G), median OS no CTx (H), median OS first-line CTx (I). Pall CTx, palliative chemotherapy; PFS, progression-free survival; CTx+bev, chemotherapy with bevacizumab; CTx, chemotherapy; F-oxali, fluoropyrimidine-oxaliplatin; F-mono, fluoropyrimidine monotherapy; OS, overall survival; F-iri, fluoropyrimidine-irinotecan; CSS, cancer-specific survival.

© 2023 de Back T et al. *JAMA Network Open.*

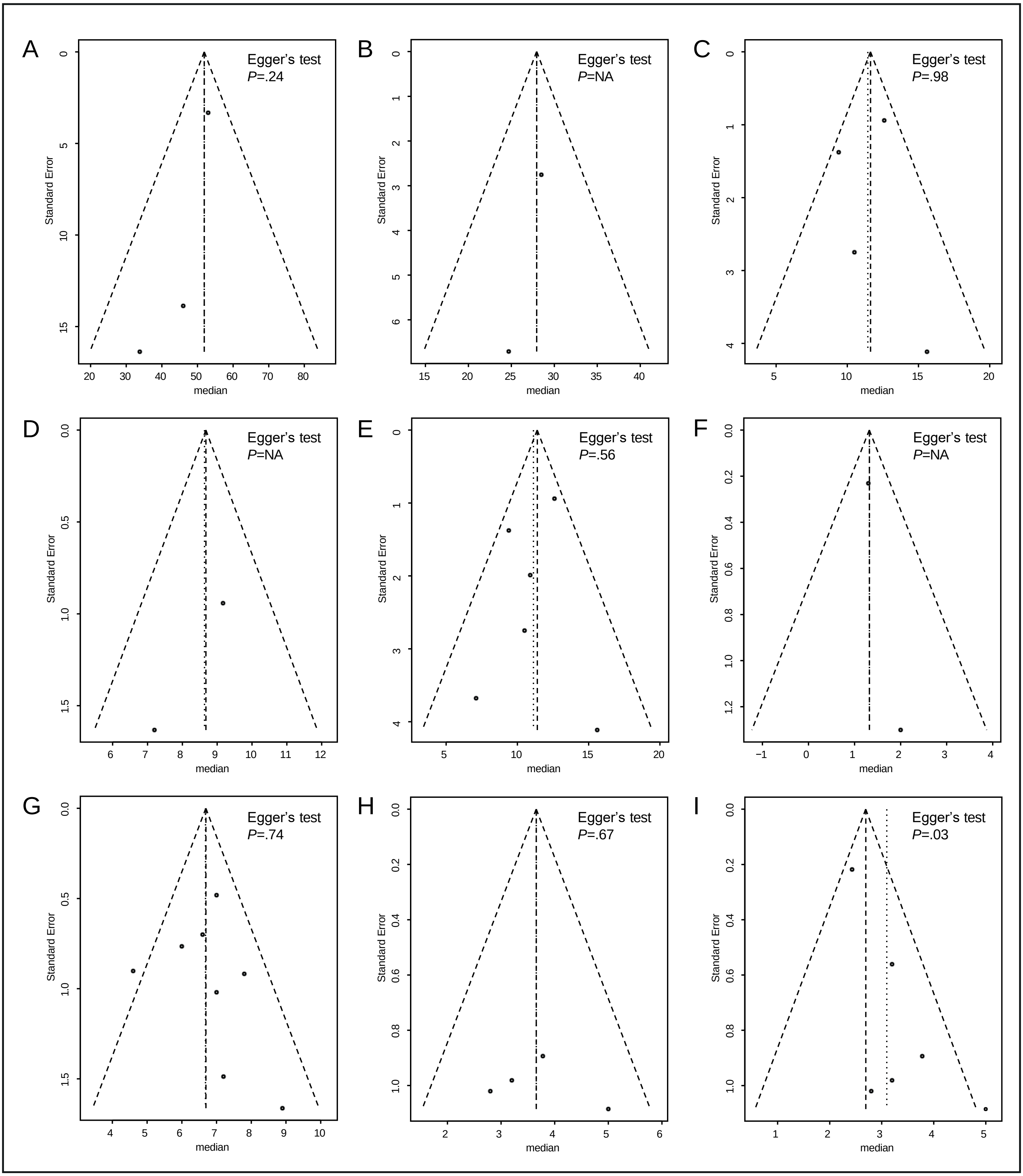

eFigure 19. Evaluation of publication bias.

Funnel plots, including Egger's test *P* values, for mOS after adj CTx (A), mOS without adj CTx (B), mOS after second-line pall CTx (C), mOS after third-line pall CTx (D), mOS after second- or third-line pall CTx (E), mPFS no pall CTx (F), mPFS first-line pall CTx (G), mPFS second-line pall CTx (H), mPFS second- and third-line pall CTx (I). mOS; median overall survival; adj CTx, adjuvant chemotherapy; pall CTx, palliative chemotherapy; mPFS, median progression-free survival.

© 2023 de Back T et al. *JAMA Network Open.*

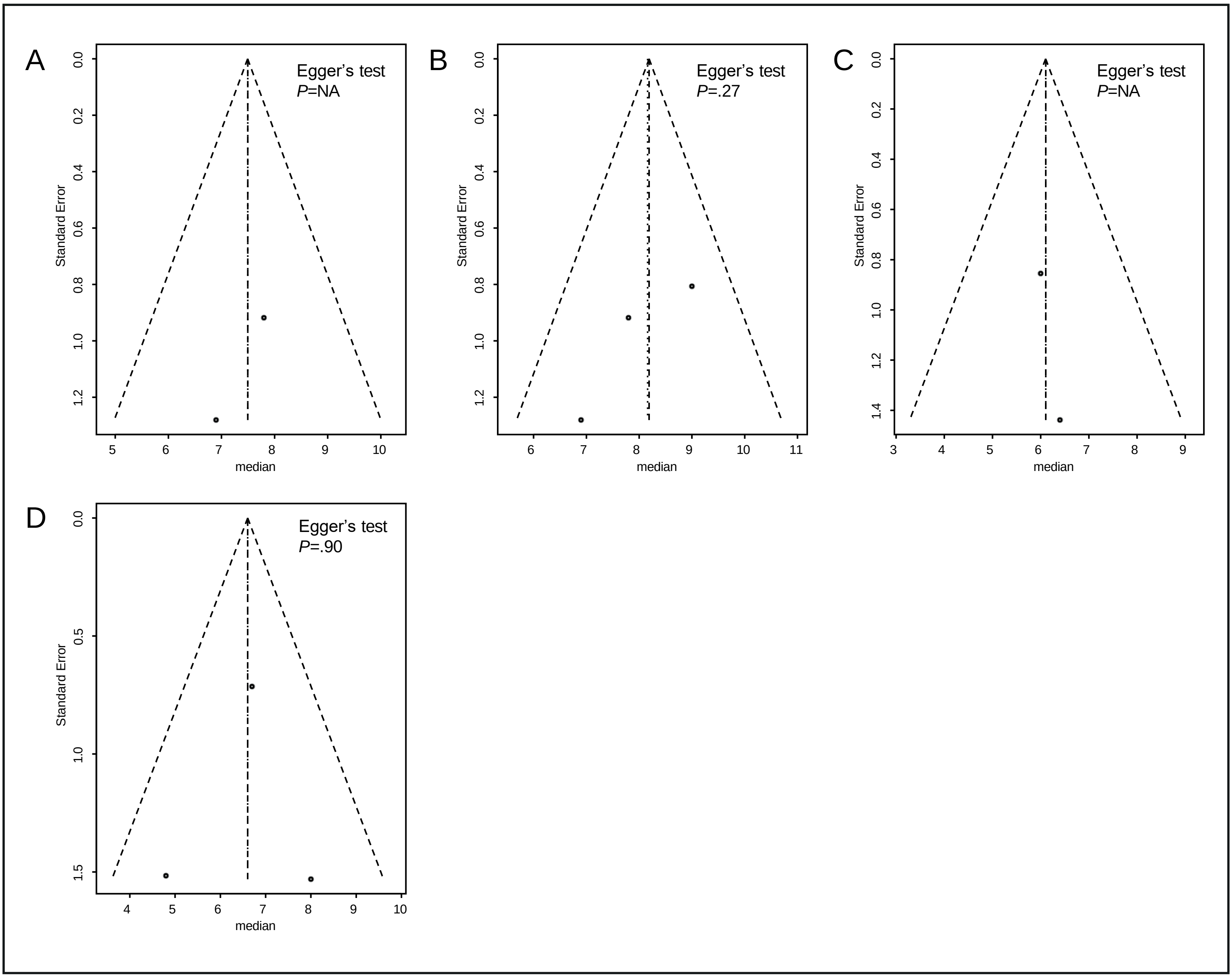

eFigure 20. Evaluation of publication bias.
Funnel plots, including Egger's test *P* values, for mPFS after pall F-oxali (A), mPFS after pall FOLFOX (B), mPFS after pall F-iri (C), and mPFS after pall F-cis (D). mPFS, median progression-free survival; pall, palliative; F-oxali, fluoropyrimidine-oxaliplatin; F-iri, fluoropyrimidine-irinotecan; F-cis, fluoropyrimidine-cisplatin; mOS, median overall survival.

eReferences

1. De Back TR NI, Sommeijer DW and Vermeulen L. PROSPERO: International Prospective Register of Systematic Reviews. 14-09-2020. https://www.crd.york.ac.uk/prospero/display_record.php?RecordID=202395
2. Ouzzani M, Hammady H, Fedorowicz Z, Elmagarmid A. Rayyan-a web and mobile app for systematic reviews. Syst Rev. Dec 5 2016;5(1):210. doi:10.1186/s13643-016-0384-4
3. Sterne JA, Hernan MA, Reeves BC, et al. ROBINS-I: a tool for assessing risk of bias in non-randomised studies of interventions. BMJ. Oct 12 2016;355:i4919. doi:10.1136/bmj.i4919
4. Altman DG, Bland JM. How to obtain the confidence interval from a P value. BMJ. Aug 8 2011;343:d2090. doi:10.1136/bmj.d2090
5. Altman DG, Bland JM. How to obtain the P value from a confidence interval. BMJ. 2011;343:d2304. doi:10.1136/bmj.d2304
6. Harrer M, Cuijpers P, Furukawa TA, Ebert DD. Doing Meta-Analysis With R: A Hands-On Guide. 1st ed. Chapman & Hall/CRC Press; 2021.
7. Shim SR, Kim SJ, Lee J, Rucker G. Network meta-analysis: application and practice using R software. Epidemiol Health. 2019;41:e2019013. doi:10.4178/epih.e2019013
8. Pollard KS, Dudoit S, van der Laan MJ. Multiple Testing Procedures: the multtest Package and Applications to Genomics. In: Gentleman R, Carey VJ, Huber W, Irizarry RA, Dudoit S, eds. Bioinformatics and Computational Biology Solutions Using R and Bioconductor. Springer New York; 2005:249-271.
9. Egger M, Davey Smith G, Schneider M, Minder C. Bias in meta-analysis detected by a simple, graphical test. BMJ. Sep 13 1997;315(7109):629-34. doi:10.1136/bmj.315.7109.629
10. Balshem H, Helfand M, Schunemann HJ, et al. GRADE guidelines: 3. Rating the quality of evidence. J Clin Epidemiol. Apr 2011;64(4):401-6. doi:10.1016/j.jclinepi.2010.07.015
11. Akce M, Jiang R, Zakka K, et al. Clinical Outcomes of Small Bowel Adenocarcinoma. Clin Colorectal Canc. 2019 2019;18(4):257-268.
12. Aparicio T, Henriques J, Manfredi S, et al. Small bowel adenocarcinoma: Results from a nationwide prospective ARCAD-NADEGE cohort study of 347 patients. Int J Cancer. 2020 2020;147(4):967-977.
13. Aparicio T, Svrcek M, Zaanan A, et al. Small bowel adenocarcinoma phenotyping, a clinicobiological prognostic study. Br J Cancer. 2013 2013;109(12):3057-3066.
14. de Jong EJM, van der Geest LG, Besselink MG, et al. Treatment and overall survival of four types of non-metastatic periampullary cancer: nationwide population-based cohort study. HPB (Oxford). Jan 23 2022;doi:10.1016/j.hpb.2022.01.009
15. Duerr D, Ellard S, Zhai Y, Taylor M, Rao S. A Retrospective Review of Chemotherapy for Patients with Small Bowel Adenocarcinoma in British Columbia. J Cancer. 2016 2016;7(15):2290-2295.
16. Ecker BL, McMillan MT, Datta J, et al. Efficacy of adjuvant chemotherapy for small bowel adenocarcinoma: A propensity score-matched analysis. Cancer. 2016 2016;122(5):693-701.
17. Fishman PN, Pond GR, Moore MJ, et al. Natural history and chemotherapy effectiveness for advanced adenocarcinoma of the small bowel: a retrospective review of 113 cases. American Journal of Clinical Oncology. 2006 2006;29(3):225-231.
18. Guo XC, Mao ZY, Su D, Wang LJ, Zhang TT, Bai L. Retrospective analysis of 119 small bowel adenocarcinoma in Chinese patients. Cancer Investigation. 2014 2014;32(5):178-183.
19. Hong SH, Koh YH, Rho SY, et al. Primary adenocarcinoma of the small intestine: presentation, prognostic factors and clinical outcome. Jpn J Clin Oncol. 2009 2009;39(1):54-61.
20. Huffman BM, Jin Z, Yadav S, et al. Novel Prognostic Factors in Resected Small Bowel Adenocarcinoma. Clin Colorectal Canc. 2019 2019;18(3):218-225.
21. Jensen KK, Storkholm JH, Chen I, Burgdorf SK, Hansen CP. Long-term results after resection of primary duodenal adenocarcinoma: A retrospective cohort study. Int J Surg. Apr 2022;100:106599. doi:10.1016/j.ijsu.2022.106599
22. Kaslow SR, Prendergast K, Vitiello GA, et al. Systemic therapy for duodenal adenocarcinoma: An analysis of the National Cancer Database (NCDB). Surgery. Jul 2022;172(1):358-364. doi:10.1016/j.surg.2022.03.009
23. Khan K, Peckitt C, Sclafani F, et al. Prognostic factors and treatment outcomes in patients with Small Bowel Adenocarcinoma (SBA): the Royal Marsden Hospital (RMH) experience. BMC Cancer. 2015 2015;15:15.
24. Kim MJ, Choi SB, Han HJ, et al. Clinicopathological analysis and survival outcome of duodenal adenocarcinoma. Kaohsiung J Med Sci. May 2014;30(5):254-9. doi:10.1016/j.kjms.2013.12.006
25. Koo DH, Yun SC, Hong YS, et al. Adjuvant chemotherapy for small bowel adenocarcinoma after curative surgery. Oncology. 2011 2011;80(3):208-213.
26. Lee TC, Wima K, Morris MC, et al. Small Bowel Adenocarcinomas: Impact of Location on Survival. Journal of Surgical Research. 2020 2020;252:116-124.

© 2023 de Back T et al. *JAMA Network Open.*

27. Legue LM, Bernards N, Gerritse SL, et al. Trends in incidence, treatment and survival of small bowel adenocarcinomas between 1999 and 2013: a population-based study in The Netherlands. Acta Oncol. Sep - Oct 2016;55(9-10):1183-1189. doi:10.1080/0284186X.2016.1182211
28. Li N, Shen W, Deng W, et al. Clinical features and the efficacy of adjuvant chemotherapy in resectable small bowel adenocarcinoma: a single-center, long-term analysis. Annals of Translational Medicine. 2020 2020;8(15):949.
29. Liang TJ, Wang BW, Liu SI, et al. Number of involved lymph nodes is important in the prediction of prognosis for primary duodenal adenocarcinoma. J Chin Med Assoc. Nov 2012;75(11):573-80. doi:10.1016/j.jcma.2012.08.002
30. Mohammed S, Anaya DA, Massarweh NN, Awad SS, Berger DH, Artinyan A. Adjuvant Chemotherapy for Adenocarcinoma of the Small Intestine in a Veteran Population: Minimal Impact on Survival. Ann Surg Oncol. 2015 2015;22(1):S177.
31. Moon YW, Rha SY, Shin SJ, Chang H, Shim HS, Roh JK. Adenocarcinoma of the small bowel at a single Korean institute: management and prognosticators. Journal of Cancer Research & Clinical Oncology. 2010 2010;136(3):387-394.
32. Nakagawa K, Sho M, Okada KI, et al. Surgical results of non-ampullary duodenal cancer: a nationwide survey in Japan. J Gastroenterol. Feb 2022;57(2):70-81. doi:10.1007/s00535-021-01841-9
33. Overman MJ, Kopetz S, Lin E, Abbruzzese JL, Wolff RA. Is there a role for adjuvant therapy in resected adenocarcinoma of the small intestine. Acta Oncol. 2010 2010;49(4):474-479.
34. Overman MJ, Kopetz S, Wen S, et al. Chemotherapy with 5-fluorouracil and a platinum compound improves outcomes in metastatic small bowel adenocarcinoma. Cancer. 2008 2008;113(8):2038-2045.
35. Platoff RM, Kellish AS, Hakim A, et al. Simple Versus Radical Resection for Duodenal Adenocarcinoma: A Propensity Score Matched Analysis of National Cancer Database. American Surgeon. 2020 2020:3134820951432.
36. Sakaguchi T, Satoi S, Hashimoto D, et al. High tumor budding predicts a poor prognosis in resected duodenal adenocarcinoma. Surg Today. Jun 2022;52(6):931-940. doi:10.1007/s00595-021-02433-z
37. Solaini L, Jamieson NB, Metcalfe M, et al. Outcome after surgical resection for duodenal adenocarcinoma in the UK. Br J Surg. May 2015;102(6):676-81. doi:10.1002/bjs.9791
38. Yanko E, Le D, Mahmood S, et al. Outcomes of Patients with Small Intestine Adenocarcinoma in a Canadian Province: A Retrospective Multi-Center Population-Based Cohort Study. Cancers (Basel). May 24 2022;14(11)doi:10.3390/cancers14112581
39. Young JI, Mongoue-Tchokote S, Wieghard N, et al. Treatment and Survival of Small-bowel Adenocarcinoma in the United States: A Comparison With Colon Cancer. Dis Colon Rectum. Apr 2016;59(4):306-15. doi:10.1097/DCR.0000000000000562
40. Zaanan A, Gauthier M, Malka D, et al. Second-line chemotherapy with fluorouracil, leucovorin, and irinotecan (FOLFIRI regimen) in patients with advanced small bowel adenocarcinoma after failure of first-line platinum-based chemotherapy. Cancer. 2011 2011;117(7):1422-1428.
41. Amano T, Iijima H, Shinzaki S, et al. Vascular endothelial growth factor-A is an Immunohistochemical biomarker for the efficacy of bevacizumab-containing chemotherapy for duodenal and jejunal adenocarcinoma. BMC Cancer. Aug 31 2021;21(1):978. doi:10.1186/s12885-021-08724-5
42. Aldrich JD, Raghav KPS, Varadhachary GR, Wolff RA, Overman MJ. Retrospective Analysis of Taxane-Based Therapy in Small Bowel Adenocarcinoma. Oncologist. 2019 2019;24(6):e384-e386.
43. Aydin D, Sendur MA, Kefeli U, et al. Evaluation of prognostic factors and treatment in advanced small bowel adenocarcinoma: report of a multi-institutional experience of Anatolian Society of Medical Oncology (ASMO). Journal of BUOn. 2016 2016;21(5):1242-1249.
44. Aydin D, Sendur MA, Kefeli U, et al. Evaluation of Bevacizumab in Advanced Small Bowel Adenocarcinoma. Clin Colorectal Canc. 2017 2017;16(1):78-83.
45. Bhamidipati D, Colina A, Hwang H, et al. Metastatic small bowel adenocarcinoma: role of metastasectomy and systemic chemotherapy. ESMO Open. Jun 2021;6(3):100132. doi:10.1016/j.esmoop.2021.100132
46. Czaykowski P, Hui D. Chemotherapy in small bowel adenocarcinoma: 10-year experience of the British Columbia Cancer Agency. Clinical Oncology (Royal College of Radiologists). 2007 2007;19(2):143-149.
47. de Jong EJM, Mommers I, Farina Sarasqueta A, et al. Adjuvant and first-line palliative chemotherapy regimens in patients diagnosed with periampullary cancer: a short report from a nationwide registry. Acta Oncol. May 2022;61(5):591-596. doi:10.1080/0284186X.2022.2053199
48. Dell'Aquila E, Zeppola T, Stellato M, et al. Anti-EGFR Therapy in Metastatic Small Bowel Adenocarcinoma: Myth or Reality? Clinical Medicine Insights Oncology. 2020 2020;14:1179554920946693.
49. Hirao M, Komori M, Nishida T, et al. Clinical use of molecular targeted agents for primary small bowel adenocarcinoma: A multicenter retrospective cohort study by the Osaka Gut Forum. Oncol Lett. 2017 2017;14(2):1628-1636.

© 2023 de Back T et al. *JAMA Network Open.*

50. Horimatsu T, Nakayama N, Moriwaki T, et al. A phase II study of 5-fluorouracil/L-leucovorin/oxaliplatin (mFOLFOX6) in Japanese patients with metastatic or unresectable small bowel adenocarcinoma. International Journal of Clinical Oncology. 2017 2017;22(5):905-912.
51. Koo DH, Yun SC, Hong YS, et al. Systemic chemotherapy for treatment of advanced small bowel adenocarcinoma with prognostic factor analysis: Retrospective study. BMC Cancer. 2011 2011;11:205.
52. Legue LM, Bernards N, Lemmens VE, de Hingh IH, Creemers GJ, van Erning FN. Palliative chemotherapy for patients with synchronous metastases of small-bowel adenocarcinoma: A reflection of daily practice. United European Gastroenterology Journal. 2019 2019;7(10):1380-1388.
53. Legue LM, Simkens GA, Creemers GJM, Lemmens VEPP, de Hingh IHJT. Synchronous peritoneal metastases of small bowel adenocarcinoma: Insights into an underexposed clinical phenomenon. Eur J Cancer. 2017 2017;87:84-91.
54. Legue LM, van Erning FN, Bernards N, Lemmens V, de Hingh I, Creemers GJ. Addition of Bevacizumab to First-Line Palliative Chemotherapy in Patients with Metastatic Small Bowel Adenocarcinoma: A Population-Based Study. Targeted Oncology. 2019 2019;14(6):699-705.
55. Liu J, Cui C, Wang J, et al. Chemotherapy and comparison of agents for advanced duodenal carcinoma. [Chinese]. Chinese Journal of Clinical Oncology. 2014 2014;41(5):319-323.
56. Liu T, Wu Y, Jiang T. Efficacy of surgery and chemotherapy for stage IV small bowel adenocarcinoma: A population-based analysis using Surveillance, Epidemiology, and End Result Program database. Cancer Med. 2020 2020;4:04.
57. McWilliams RR, Foster NR, Mahoney MR, et al. North Central Cancer Treatment Group N0543 (Alliance): A phase 2 trial of pharmacogenetic-based dosing of irinotecan, oxaliplatin, and capecitabine as first-line therapy for patients with advanced small bowel adenocarcinoma. Cancer. 2017 2017;123(18):3494-3501.
58. Nakazawa T, Narita Y, Kumanishi R, et al. P-163 Systemic chemotherapy for previously treated metastatic small bowel adenocarcinoma. Ann Oncol. 2020 2020;31:S143.
59. Overman MJ, Adam L, Raghav K, et al. Phase II study of nab-paclitaxel in refractory small bowel adenocarcinoma and CpG island methylator phenotype (CIMP)-high colorectal cancer. Ann Oncol. 2018 2018;29(1):139-144.
60. Pedersen KS, Foster NR, Overman MJ, et al. ZEBRA: A Multicenter Phase II Study of Pembrolizumab in Patients with Advanced Small-Bowel Adenocarcinoma. Clin Cancer Res. Jul 1 2021;27(13):3641-3648. doi:10.1158/1078-0432.CCR-21-0159
61. Takayoshi K, Kusaba H, Uenomachi M, et al. Suggestion of added value by bevacizumab to chemotherapy in patients with unresectable or recurrent small bowel cancer. Cancer Chemotherapy & Pharmacology. 2017 2017;80(2):333-342.
62. Tsushima T, Taguri M, Honma Y, et al. Multicenter retrospective study of 132 patients with unresectable small bowel adenocarcinoma treated with chemotherapy. Oncologist. 2012 2012;17(9):1163-1170.
63. Xiang XJ, Liu YW, Zhang L, et al. A phase II study of modified FOLFOX as first-line chemotherapy in advanced small bowel adenocarcinoma. Anti-Cancer Drugs. 2012 2012;23(5):561-566.
64. Ye X, Wang L, Xing Y, Song C. Frequency, prognosis and treatment modalities of newly diagnosed small bowel cancer with liver metastases. BMC Gastroenterology. 2020 2020;20(1)
65. Yhim HY, Cho SH, Kim SY, et al. Prognostic implications of thymidylate synthase gene polymorphisms in patients with advanced small bowel adenocarcinoma treated with first-line fluoropyrimidine-based chemotherapy. Oncology Reports. 2015 2015;34(1):155-164.
66. Zaanan A, Costes L, Gauthier M, et al. Chemotherapy of advanced small-bowel adenocarcinoma: a multicenter AGEO study. Ann Oncol. 2010 2010;21(9):1786-1793.
67. Zhu H, Zhao S, Zhao T, et al. Development and validation of prognostic nomograms for patients with metastatic small bowel adenocarcinoma: a retrospective cohort study. Sci Rep. Apr 8 2022;12(1):5983. doi:10.1038/s41598-022-09986-0

© 2023 de Back T et al. *JAMA Network Open.*
