# Supplementary material for: Evaluation of Systemic Treatments of Small Intestinal Adenocarcinomas: A Systematic Review and Meta-analysis
Source: JAMA Netw Open. 2023 Feb 24;6(2):e230631. doi: 10.1001/jamanetworkopen.2023.0631 (PMC9958532; doi:10.1001/jamanetworkopen.2023.0631)
Supplement: Supplement 2. — Data Sharing Statement [file jamanetwopen-e230631-s002.pdf]

Data Sharing Statement

de Back. Evaluation of Systemic Treatments of Small Intestinal Adenocarcinomas. *JAMA Netw Open*. Published February 24, 2023. doi:10.1001/jamanetworkopen.2023.0631

Data
Data available: No

Additional Information
Explanation for why data not available: Data in the manuscript was obtained from original publications. Data could be requested from the authors of the original publications.
